# Auditory cortex ensembles jointly encode sound and locomotion speed to support sound perception during movement

**DOI:** 10.1371/journal.pbio.3002277

**Published:** 2023-08-31

**Authors:** Carlos Arturo Vivaldo, Joonyeup Lee, MaryClaire Shorkey, Ajay Keerthy, Gideon Rothschild

**Affiliations:** 1 Department of Psychology, University of Michigan, Ann Arbor, Michigan, United States of America; 2 Kresge Hearing Research Institute and Department of Otolaryngology—Head and Neck Surgery, University of Michigan, Ann Arbor, Michigan, United States of America; Universidad de Salamanca, SPAIN

## Abstract

The ability to process and act upon incoming sounds during locomotion is critical for survival and adaptive behavior. Despite the established role that the auditory cortex (AC) plays in behavior- and context-dependent sound processing, previous studies have found that auditory cortical activity is on average suppressed during locomotion as compared to immobility. While suppression of auditory cortical responses to self-generated sounds results from corollary discharge, which weakens responses to predictable sounds, the functional role of weaker responses to unpredictable external sounds during locomotion remains unclear. In particular, whether suppression of external sound-evoked responses during locomotion reflects reduced involvement of the AC in sound processing or whether it results from masking by an alternative neural computation in this state remains unresolved. Here, we tested the hypothesis that rather than simple inhibition, reduced sound-evoked responses during locomotion reflect a tradeoff with the emergence of explicit and reliable coding of locomotion velocity. To test this hypothesis, we first used neural inactivation in behaving mice and found that the AC plays a critical role in sound-guided behavior during locomotion. To investigate the nature of this processing, we used two-photon calcium imaging of local excitatory auditory cortical neural populations in awake mice. We found that locomotion had diverse influences on activity of different neurons, with a net suppression of baseline-subtracted sound-evoked responses and neural stimulus detection, consistent with previous studies. Importantly, we found that the net inhibitory effect of locomotion on baseline-subtracted sound-evoked responses was strongly shaped by elevated ongoing activity that compressed the response dynamic range, and that rather than reflecting enhanced “noise,” this ongoing activity reliably encoded the animal’s locomotion speed. Decoding analyses revealed that locomotion speed and sound are robustly co-encoded by auditory cortical ensemble activity. Finally, we found consistent patterns of joint coding of sound and locomotion speed in electrophysiologically recorded activity in freely moving rats. Together, our data suggest that rather than being suppressed by locomotion, auditory cortical ensembles explicitly encode it alongside sound information to support sound perception during locomotion.

## Abstract 2

A sound can hold different behavioral relevance depending on the locomotive state, requiring the brain to integrate incoming sounds with the body’s locomotion. This study identifies a novel neural population code in the auditory cortex of rodents which integrates external sounds with the animal’s locomotion speed.

## Introduction

Continuous processing of incoming sensory information is critical for survival and adaptive behavior. While the neural mechanisms of sensory processing have traditionally been studied in immobile subjects, some of the most critical behaviors in humans and other animal species, such as foraging for food, seeking a mate, and navigating to safety, occur during locomotion. To gain a coherent perception of the environment during locomotion and be able to rapidly trigger appropriate behavior, the brain must encode incoming external cues and integrate them with one’s own motion. For example, humans integrate incoming sounds with locomotion during simple walking, as manifested by the modification of walking pace based on auditory feedback [1–6]. Moreover, auditory feedback has been shown to improve walking in aged patients and those with neurodegenerative disorders [7–9]. Integration of sounds with self-motion has also been studied in the context of other behaviors such as dance [10,11] and sound-guided finger tapping [12–15]. In nonhumans, perhaps the best known example is bat echolocation [16–18], yet various forms of audiomotor integration have been studied in diverse animal species, including praying mantids [19], dholes [20], and mice [21]. Thus, the ability to process incoming sounds during locomotion and integrate them with the locomotive state to guide appropriate behavior is fundamental in both humans and other animal species.

The auditory cortex (AC) is a key candidate brain region for processing incoming sounds during locomotion due to its well-established role in context-, behavior-, and decision-making-dependent sound processing [22–34]. Intriguingly, previous studies have found that locomotion has a generally suppressive effect on sound-evoked responses in the AC [35–39]. Attenuation of responses to self-generated sounds produced during locomotion is well explained by corollary discharge, which acts to suppress responses to predictable sounds and enhance sensitivity to unpredictable sounds [38,40–43] (though see [44–46]). However, the functional benefit of the observed attenuation of AC responses to unpredictable external sounds during locomotion has remained elusive. A proposed explanation, supported by the finding that responses in the primary visual cortex are generally enhanced during locomotion [47–49], is that locomotion reflects a neural resource allocation shift from audition to vision [35,36]. According to this model, reduced sound responses during locomotion reflect a functional attenuation of AC, possibly involving reliance on subcortical regions for sound processing in this state. However, the evolutionary and functional benefit of this suggestion remains debated [37]. Alternatively, it has been suggested that, rather than a resource allocation shift from the auditory to visual modality, locomotion may induce a shift towards spatial information processing across modalities [37]. According to this hypothesis, the AC may play a critical role in sound processing during locomotion, but that it may include encoding of locomotion-related non-acoustic information, which, if unaccounted for, could appear as simple suppression. This hypothesis is supported by robust encoding of locomotion-related signals and their integration with cue-evoked responses in other sensory cortical regions [50–54]. However, this hypothesis has yet to be directly tested in the AC. Here, we combined AC inactivation in mice performing sound-guided reward-predictive behavior during locomotion, two-photon calcium imaging in head-fixed mice, and electrophysiological recordings in freely moving rats to test the hypothesis that auditory cortical ensembles are not simply suppressed during locomotion but rather explicitly encode it and incorporate it with sound information into an integrated audiomotor neural code.

## Results

### Auditory cortical activity is required for sound processing during locomotion

**Fig 1:**
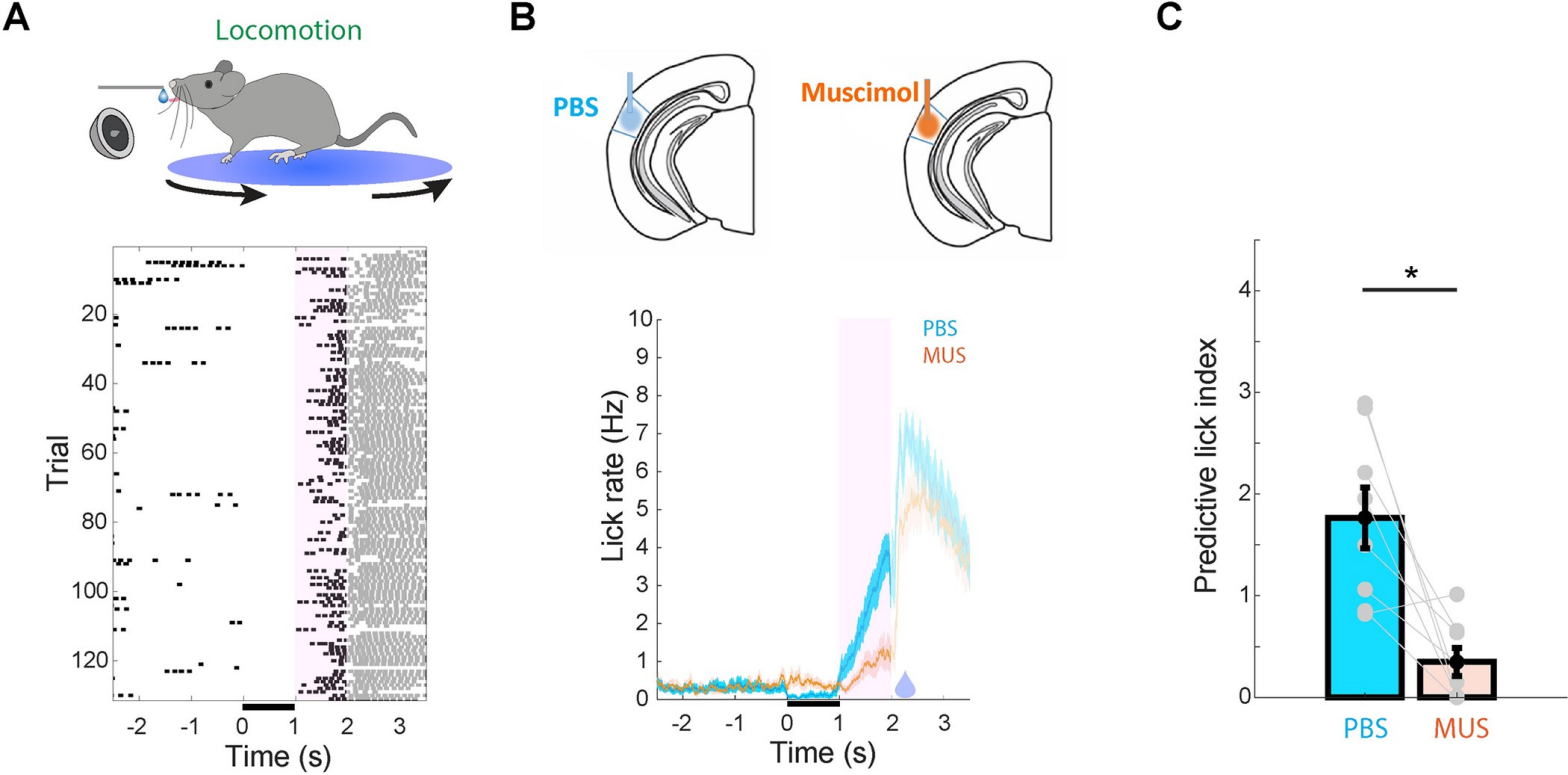
Auditory cortical activity is necessary for sound processing during locomotion. **(A)** Top: Illustration of the behavioral setup for sound-guided predictive licking in locomotion. Bottom: Peri-sound lick raster of an example behavioral session from a trained animal performing the task. Licking in the pink shaded area following sound termination represents prediction of upcoming reward (delivered at 2 s). Licks following reward delivery are shaded as they do not require sound processing or reward prediction **(B)** Peri-sound lick histogram across animals performing the task when the AC was infused with either PBS or muscimol. Solid lines denote the mean and the shaded area represents SEM across animals. Predictive licking is reduced following AC inactivation using muscimol. **(C)** There was a significant reduction in predictive lick index following infusion of MUS (*P* = 0.0156, signed-rank test). Error bars represent mean ± SEM across animals. Lines connecting gray circles represent data from the same animal in the different conditions. The data underlying this figure can be found in Fig 1 data at https://doi.org/10.6084/m9.figshare.23736831. AC, auditory cortex; MUS, muscimol; PBS, phosphate buffer solution.

Previous studies have shown that during immobility, the AC is not necessary for simple tone detection or discrimination, but is required for more demanding tasks such as discrimination of complex sounds and sound source localization [55–64] (though see [65]). To determine whether AC activity is required for sound-guided behavior during locomotion, we measured the influence of AC inactivation on sound-guided reward-predictive licking during locomotion in mice. Male and female mice were first implanted with bilateral cannula into the AC for subsequent drug delivery and allowed to recover for at least 5 days. Mice were then put on water restriction and were trained on an appetitive trace conditioning task during head fixation while standing on a rotatable plate that allowed the animals to stand or run at will. Using a closed-loop system that received the output of a rotary encoder at the base of the plate, training trials were selectively initiated during locomotion and consisted of the presentation of an 8 kHz tone followed by a drop of water reward, delivered 1 s after sound termination. Mice (*n* = 8) were trained until they learned the sound-reward association as evidenced by an increase in lick rate following the sound and before reward delivery (“predictive licking,” **Fig 1A**). To test whether AC activity is necessary for this behavior, we measured the influence of AC inactivation on sound-triggered reward-predictive licking. To this end, we measured behavioral performance in trained mice following infusion of the GABAA receptor agonist muscimol (MUS), or inert phosphate buffer solution (PBS) as a control, into the AC, in a within-subject design (**Fig 1B**). We found that inactivation of the AC induced a significant and near-complete reduction in sound-triggered predictive licking during locomotion (**Fig 1B and 1C**). Furthermore, while following PBS delivery mice exhibited sound-triggered reduction of running speed leading to the time of reward delivery, this effect was significantly weaker following MUS delivery (**S1 Fig**). In a version of this task performed during immobility, AC inactivation induced a trend of an impairment, but it did not reach significance (**S2 Fig**), consistent with previous studies [55,57–59,66].

The finding that during locomotion, the AC plays an important role in sound-guided predictive licking and locomotion speed modulation suggests that its reduced sound-evoked responses during locomotion may reflect part of an alternative neural computation in this state. To test this possibility, we carried out optical recording of AC ensemble activity in mice.

### A heterogeneous and overall inhibitory influence of locomotion on sound-evoked responses of local excitatory L2/3 neuronal ensembles in the auditory cortex

**Fig 2:**
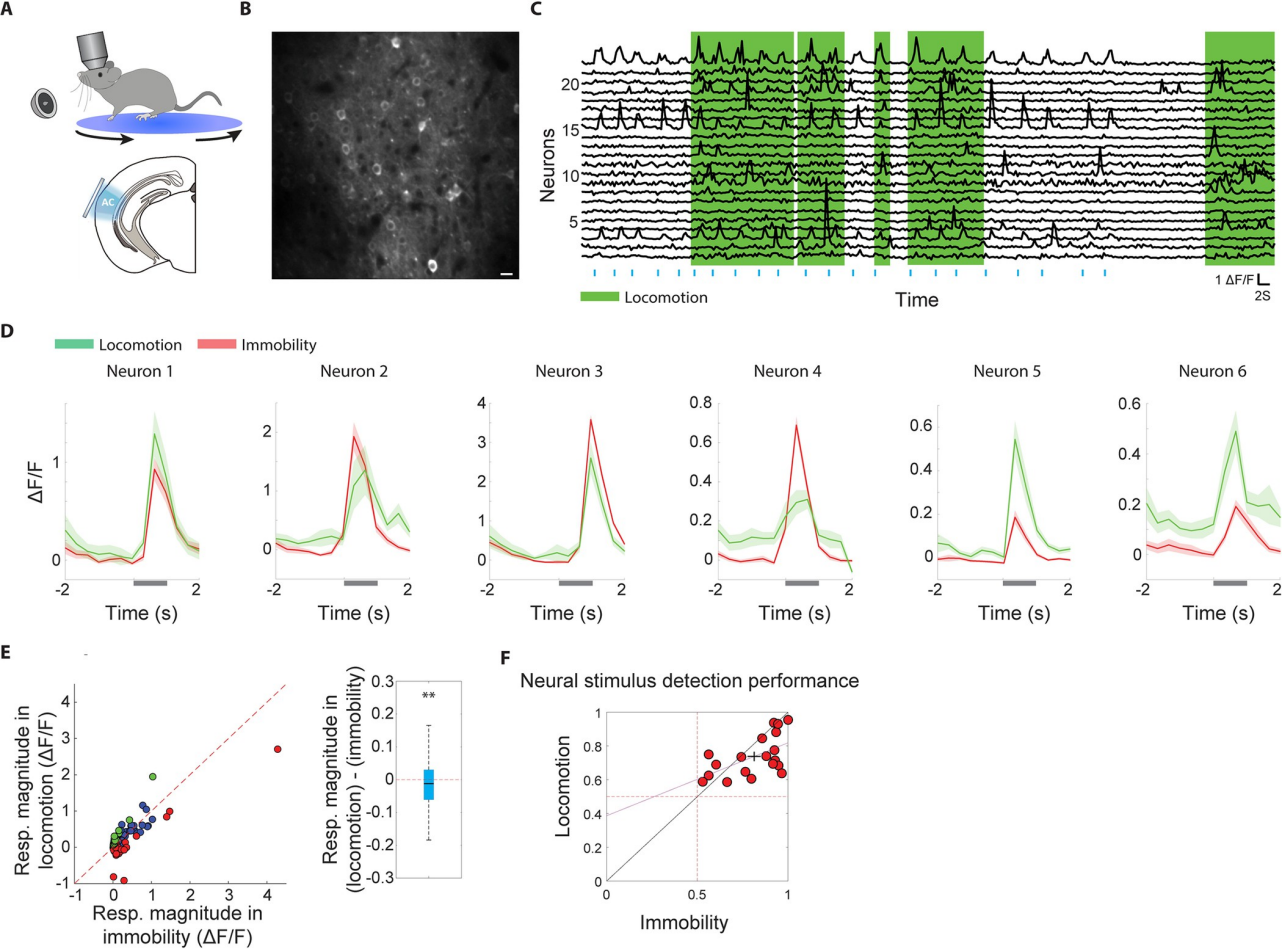
A heterogeneous and overall inhibitory influence of locomotion on sound-evoked responses of local excitatory L2/3 neuronal ensembles in the AC. **(A)** Illustration of the experimental setup. **(B)** Two-photon average micrograph of an example local neuronal ensemble in L2/3 of the AC. Scale bar: 10 μm. **(C)** Relative change in fluorescence (ΔF/F) of 22 neurons from the micrograph in “B” during an imaging session. Periods of locomotion are marked in green. **(D)** Sound-triggered PSTHs from 6 example neurons. Sound presentation trials in which the animal was immobile (red) and running (green) were grouped separately. Locomotion had diverse effects on sound-evoked responses of different neurons, including invariance (neurons 1+2), reduction (neurons 3+4), and enhancement (neurons 5+6). **(E)** Left: Sound-evoked responses in immobility and locomotion across all BBN-responsive neurons. Red and green dots represent neurons that individually exhibited a significantly stronger and weaker response during immobility, respectively. Blue dots represent neurons that did not exhibit a significant difference. Right: Box plot describing sound-evoked responses in locomotion minus immobility across all BBN-responsive neurons. The distribution was significantly lower than 0 (*P* = 0.009, two-sided Wilcoxon signed-rank). For this and subsequent whisker plots, the central mark indicates the median, the bottom and top edges of the box indicate the 25th and 75th percentiles, respectively, and the whiskers extend to the most extreme data points not considered outliers. **(F)** Ensemble-level neural stimulus detection performance in immobility and locomotion. Detection performance in immobility and locomotion was significantly correlated across ensembles (*P* = 0.012, Pearson correlation). Detection in immobility was significantly higher than in locomotion (*P* = 0.036, signed-rank test). The data underlying this figure can be found in Fig 2 data at https://doi.org/10.6084/m9.figshare.23736831. AC, auditory cortex; BBN, broadband noise.

To study the nature of information processing by local groups of L2/3 excitatory neurons (“neuronal ensembles”) of the AC during locomotion, we carried out two-photon calcium imaging in head-fixed Thy1-GCaMP6f mice that were free to stand or run at will on a rotatable plate (**Fig 2A–2C**). We first examined how locomotion modulates the responses of neurons to broadband noise (BBN) bursts in 985 AC neurons from 7 mice, of which 612 neurons had a sufficient number of responses in both immobility and locomotion to allow for comparison. In keeping with most previous studies, we started with examining baseline-subtracted responses, which are defined as the difference between the activity evoked by the sound and the activity immediately preceding the sound. Locomotion had a diverse influence on sound-evoked responses of individual neurons, including invariance, suppression, and enhancement (**Fig 2D**). Across all neurons that exhibited significant BBN-evoked responses in immobility (194/612, 31.7%, of which sound-evoked response magnitudes of 23/194, 34/194, and 137/194 were individually significantly enhanced, suppressed, and not showing a significant difference, respectively), the population-average responses were significantly reduced during locomotion (**Fig 2E**), consistent with previous studies [35–37]. To test whether these findings were unique to responses to BBN, we examined how locomotion modulates responses to pure tones and complex sounds. These experiments revealed a similar influence of locomotion on sound-evoked responses, namely a net population-average decrease that coexists with heterogeneous influences at the single-cell level (**S3 Fig**).

Given our observation that sound-evoked responses of individual neurons showed heterogeneous modulation by locomotion, and that across animals the location of the imaging field within AC may slightly vary, we wondered whether some local groups of neurons were preferentially dedicated to sound processing during locomotion and others to sound processing in immobility. To test this, we used cross-validated classification models to quantify ensemble-level stimulus detection in immobility and locomotion (separately) for each ensemble. We posited that if sound detection in immobility and locomotion is supported by distinct ensembles, stimulus detection performance in immobility and locomotion would be negatively correlated across ensembles. Instead, we found that stimulus detection performance in locomotion and immobility were significantly positively correlated (**Fig 2F**). Furthermore, this analysis showed that across ensembles, stimulus detection performance was mildly but significantly lower in locomotion as compared to immobility, consistent with the average weaker sound-evoked responses in locomotion (**Fig 2E**). Thus, overlapping AC L2/3 ensembles encode sounds during immobility and locomotion, with a net weaker sound detection performance during locomotion.

### Enhanced ongoing activity during locomotion reduces baseline-subtracted sound-evoked response magnitudes

**Fig 3:**
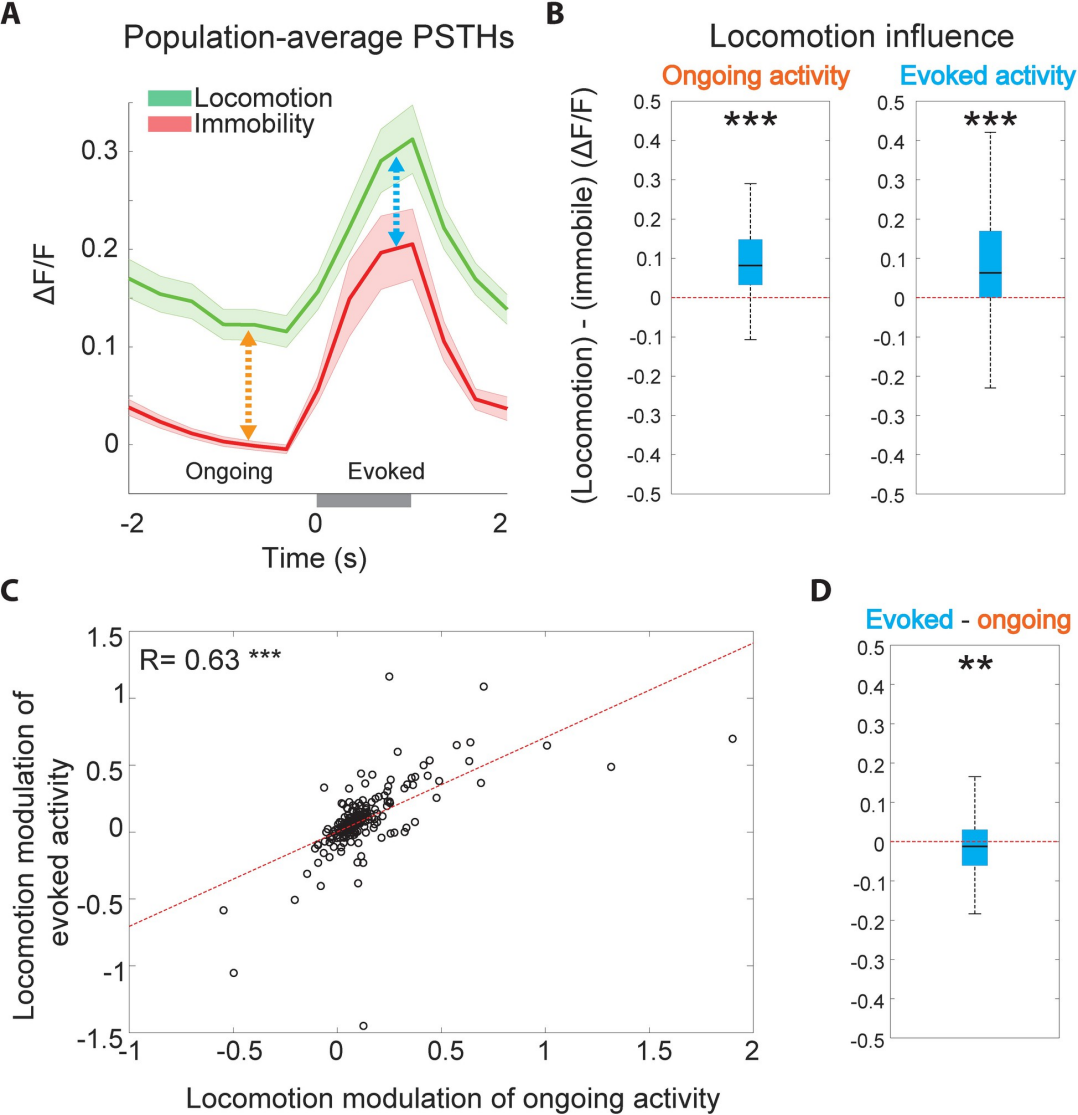
Enhanced ongoing activity during locomotion reduces baseline-subtracted sound-evoked responses. **(A)** Population-level PSTH across all BBN-responsive neurons during immobility (red) and locomotion (green). Solid lines and shaded areas indicate mean ± SEM. **(B)** Locomotion increased ongoing activity of sound-responsive neurons (left, *P* = 2.9 × 10^−23^, two-sided Wilcoxon signed-rank test), as well as of evoked activity (right, *P* = 8 × 10^−13^, two-sided Wilcoxon signed-rank test). **(C)** Locomotion influence on ongoing and evoked activity across neurons were significantly correlated. **(D)** The per-neuron difference between the locomotion influence on evoked and ongoing activity. The locomotion influence on evoked activity was significantly lower than that of ongoing activity, resulting in a net reduction in baseline-subtracted sound-evoked responses (*P* = 0.0094, two-sided Wilcoxon signed-rank test). The data underlying this figure can be found in Fig 3 data at https://doi.org/10.6084/m9.figshare.23736831. BBN, broadband noise; PSTH, peri-stimulus time histogram.

We sought to further investigate the source of the net reduction in baseline-subtracted sound-evoked responses and noticed that many neurons exhibited increased ongoing activity during locomotion, which manifested as increased activity before stimulus onset (**Fig 2D**). Increased baseline activity during locomotion could contribute to reduced sound responses by increasing the subtrahend in the baseline-subtracted sound-evoked response calculation. To test this possibility, we calculated the average sound-triggered peri-stimulus time histogram (PSTH) across the population of BBN-responsive neurons and found that it exhibits a significant elevation in ongoing, pre-stimulus activity during locomotion as compared to immobility (**Fig 3A**, orange arrow, **Fig 3B**, left panel). Increased ongoing activity during locomotion was also observed in the presence of a constant masking sound, suggesting it is at least partly independent of self-generated sounds (**S4 Fig**). Locomotion also produced a significant increase in evoked activity during the stimulus time window (**Fig 3A**, blue arrow, **Fig 3B**, right panel), and the locomotion influences on ongoing and evoked activity were positively correlated across neurons (**Fig 3C**). However, the locomotion-induced increase in activity in the evoked window was significantly smaller than the increase in ongoing activity, likely reflecting a saturation effect, resulting in a net negative influence of locomotion on baseline-subtracted sound-evoked activity (**Fig 3D**). This suggests that increased ongoing activity during locomotion compresses the dynamic range of the baseline-subtracted response. When including sound-unresponsive cells, locomotion increased activity during the ongoing and sound time windows, but did not induce a significant reduction in baseline-subtracted sound-evoked responses, as expected (**S5 Fig**).

These data demonstrate that the observed average reduction in baseline-subtracted sound responses during locomotion is at least partly due to increased ongoing, pre-sound activity. We therefore wondered whether this enhanced ongoing activity during locomotion, which is subtracted out in the standard calculation of sound response magnitude and impairs neural sound detection, may in fact reflect encoding of meaningful information for auditory cortical processing during behavior.

### Enhanced ongoing activity reliably encodes locomotion speed

**Fig 4:**
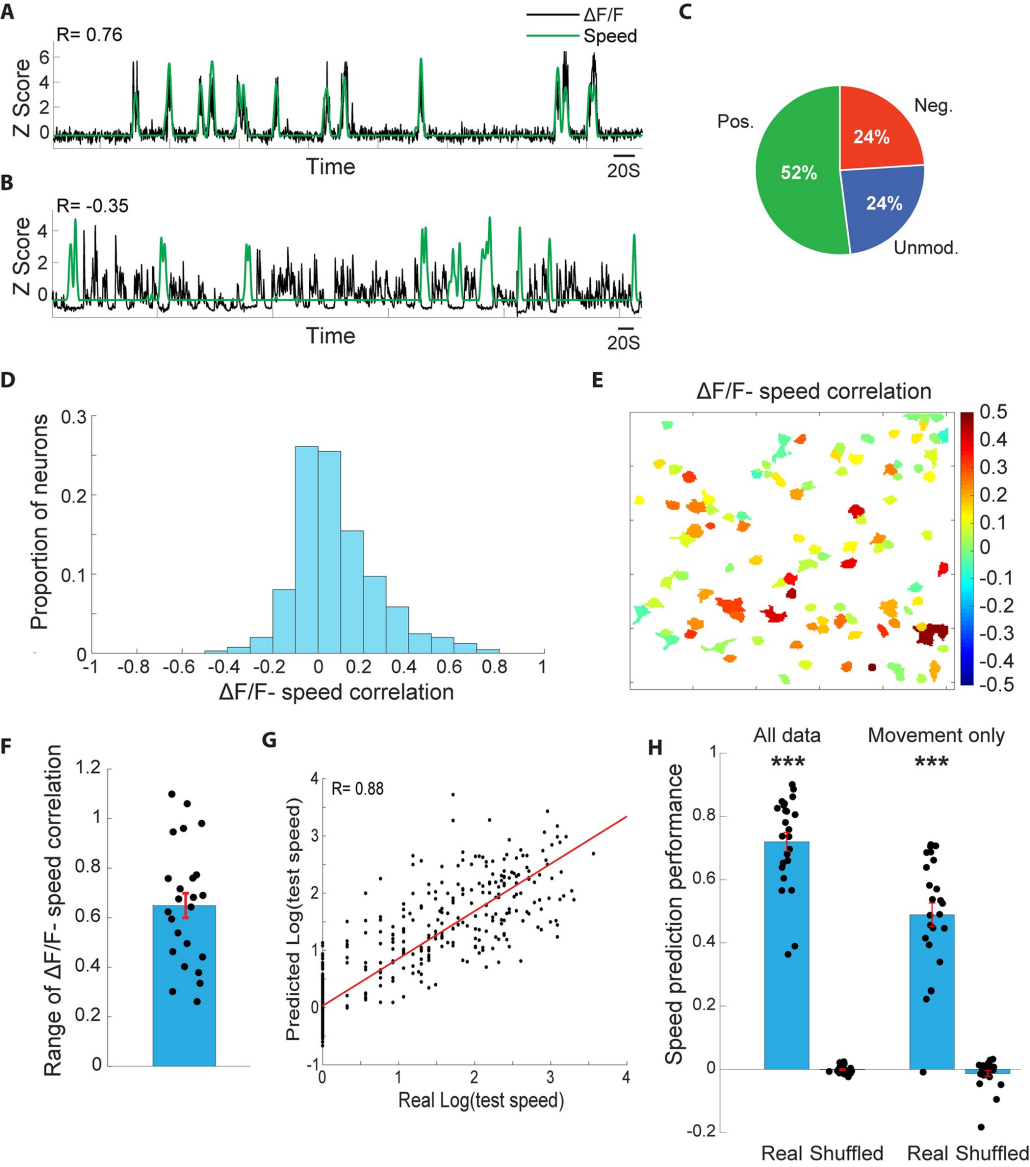
Auditory cortical L2/3 neurons and ensembles reliably encode locomotion speed. **(A)** Z-scored ΔF/F of an example neuron (black trace) overlaid on the Z-scored locomotion speed of the mouse (green trace) during an example imaging session. This neuron exhibited a correlation of R = 0.76 with locomotion speed across the session. **(B)** An example from a different neuron, showing a negative correlation with locomotion speed of R = −0.35. **(C)** Proportions of AC L2/3 neurons showing significant positive, significant negative, and nonsignificant correlation with locomotion speed. **(D)** The distribution of ΔF/F-locomotion speed correlations across the population. **(E)** An illustration of all neurons in an example imaging session, color coded according to each neuron’s ΔF/F-locomotion speed correlation value. Local ensembles exhibited a high degree of heterogeneity in correlation with locomotion speed. **(F)** The ensemble-level range in ΔF/F-locomotion speed correlation values across ensembles. **(G)** The predicted log (speeds) of an example test set against the real log(speeds) of that test set, showing a correlation of 0.88. **(H)** Speed prediction performance, measured as the correlation values between the predicted and real locomotion speeds across ensembles. Shuffled values were derived by randomly shuffling the predicted speed values. Left: all data included (*P* = 3 × 10^−9^), right: movement-only (*P* = 1.9 × 10^−8^). The data underlying this figure can be found in Fig 4 data at https://doi.org/10.6084/m9.figshare.23736831. AC, auditory cortex.

During locomotion, a key behavioral parameter that can shape how to process and act upon incoming sensory stimuli is locomotion speed [52]. In particular, robust speed coding in the hippocampus and medial entorhinal cortex are believed to be critical for cue-guided navigation [67–71]. Moreover, hippocampal coding of space and locomotion is coordinated with the primary visual cortex [54,72], where locomotion speed is robustly encoded and integrated with cue-evoked responses [50,53]. We therefore tested the hypothesis that the enhanced ongoing activity that we observed during locomotion encodes movement speed. To test this hypothesis, we first asked whether neural activity of individual neurons is significantly correlated with locomotion speed. We calculated the correlations between the continuous relative change in fluorescence of each neuron and the running speed of the mouse, utilizing a large subset of our imaged neurons (647/985) that were imaged while the continuous running speed of the animal was acquired. We found that activity of auditory cortical neurons could exhibit surprisingly high positive correlations with locomotion speed (**Fig 4A**), and in fewer cases significant negative correlations with locomotion speed (**Fig 4B**). Across the population, ongoing activity of 52% of neurons (335/647) showed significant positive correlation with locomotion speed, 24% of neurons (155/647) exhibited significant negative correlation with locomotion speed, and 24% (157/647) showed no significant correlation with locomotion speed (**Fig 4C**). The distribution of correlations between neural activity and locomotion speed was skewed to the right (**Fig 4D,** skewness = 0.84), consistent with our finding of a population-level enhancement in baseline activity during locomotion.

Interestingly, we also found that within local excitatory auditory cortical ensembles in L2/3, individual neurons exhibited high diversity in correlations between neural activity and locomotion speed (**Fig 4E**). Within local neuronal ensembles, the average range of correlations between locomotion speed and relative change in fluorescence of the different neurons was 0.65 (**Fig 4F**). These findings suggest that, similar to the heterogeneous local organization of sound responses in the AC [57,73–80], locomotion modulates ongoing activity of local excitatory neuronal populations in a spatially fine-tuned manner rather than acting as a global uniform modulator.

To further quantify the degree of information that auditory cortical ensembles convey about locomotion speed, we implemented a cross-validated generalized linear model (GLM) to test if locomotion speed can be decoded from ongoing ensemble activity. For each imaging session of a single neuronal ensemble, a GLM was trained on a random half of the imaging session data and tested on the other half, and this procedure was repeated 200 times for robust estimation. In the test phase, the GLM model that was constructed in the training phase predicted locomotion speed based on ensemble patterns of neural activity of the test set. We found that in many cases, the predictions of the model were highly correlated with the actual speeds (**Fig 4G**). The correlations between the predicted speed and real speed were large and highly significant, even when excluding all immobility periods (**Fig 4H**). These findings suggest that ongoing locomotion speed is reliably encoded by the activity of local neuronal ensembles in the AC.

### Integration of sound and locomotion information by excitatory neuronal ensembles in L2/3 of the auditory cortex

Taken together, our results suggest that excitatory neurons in L2/3 of the AC robustly encode both external sounds and locomotion speed. These findings raise the question of whether these 2 variables—external sounds and locomotion—are simultaneously represented and integrated within the local network level.

**Fig 5:**
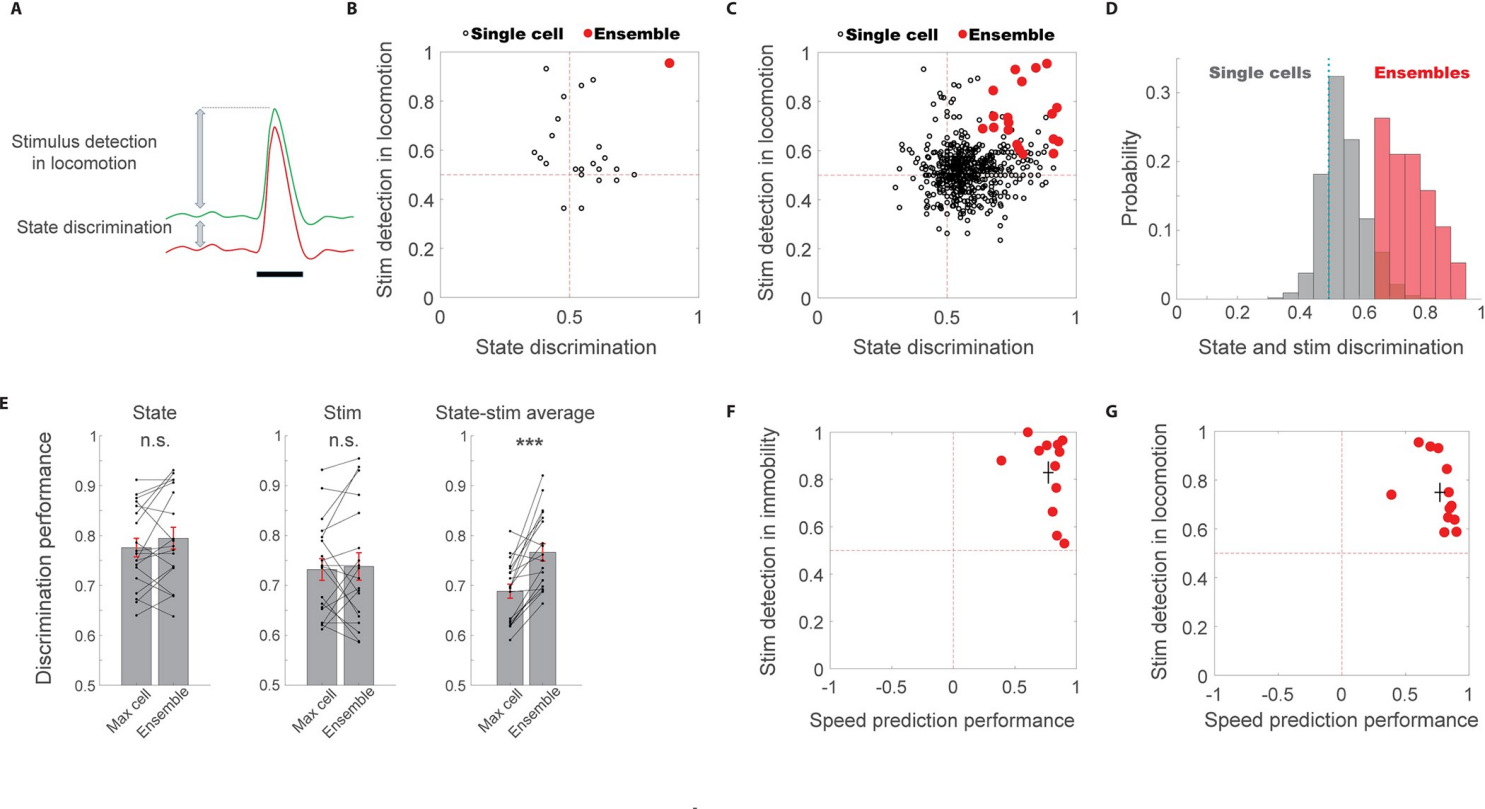
Integration of sound and locomotion information by excitatory neuronal ensemble in L2/3 of the AC. **(A)** Schematic illustration of the measures used for stimulus detection in locomotion and state discrimination. **(B)** Performance of stimulus detection in locomotion against state discrimination in an example ensemble. **(C)** Performance of stimulus detection in locomotion against state discrimination across ensembles and single cells. **(D)** Histogram of average state and stim discrimination for single cells (gray) and ensembles (red). **(E)** Comparison of discrimination performance of ensembles and their best-predictive neuron (per attribute), for State (left, *P* = 0.21), Stim (middle, *P* = 0.687), and State-stim average (right, *P* = 0.0004), signed-rank test. **(F)** Stimulus detection in immobility against speed prediction performance across ensembles. Black cross shows mean ± STD of the 2 measures. **(G)** Stimulus detection in locomotion against speed prediction performance across ensembles. The data underlying this figure can be found in Fig 5 data at https://doi.org/10.6084/m9.figshare.23736831. AC, auditory cortex.

To test this, we first quantified whether the activity of individual neurons and local ensembles could predict both locomotion state (immobility/locomotion) and sound occurrence during locomotion (*n* = 19 ensembles). To this end, we implemented cross-validated support vector machine (SVM) analyses on each neuron’s or ensemble’s activity patterns and quantified the predictive power that it provided to discriminate between immobility and locomotion and between sound occurrence and no sound during locomotion (**Fig 5A**). We found that while individual neurons typically showed moderate prediction, with high prediction of at most one of these attributes, local ensembles could display high prediction of both locomotion state and stimulus occurrence (**Fig 5B–5D**). Indeed, while discrimination performance of ensembles was not better than that of their best-predicting individual neuron for sound or state, sound-state discrimination average was significantly higher at the ensemble level than the best neuron, demonstrating sound-state integration at the ensemble level (**Fig 5E**). Furthermore, we found that local neuronal ensembles consisting of a few dozen neurons could exhibit both high-fidelity speed coding and stimulus detection in both immobility (**Fig 5F**) and locomotion (**Fig 5G**). Together, these data suggest that neuronal ensembles in L2/3 of the AC robustly co-encode locomotion speed alongside sound information during movement.

### Integration of sound and locomotion in the freely moving rat

**Fig 6:**
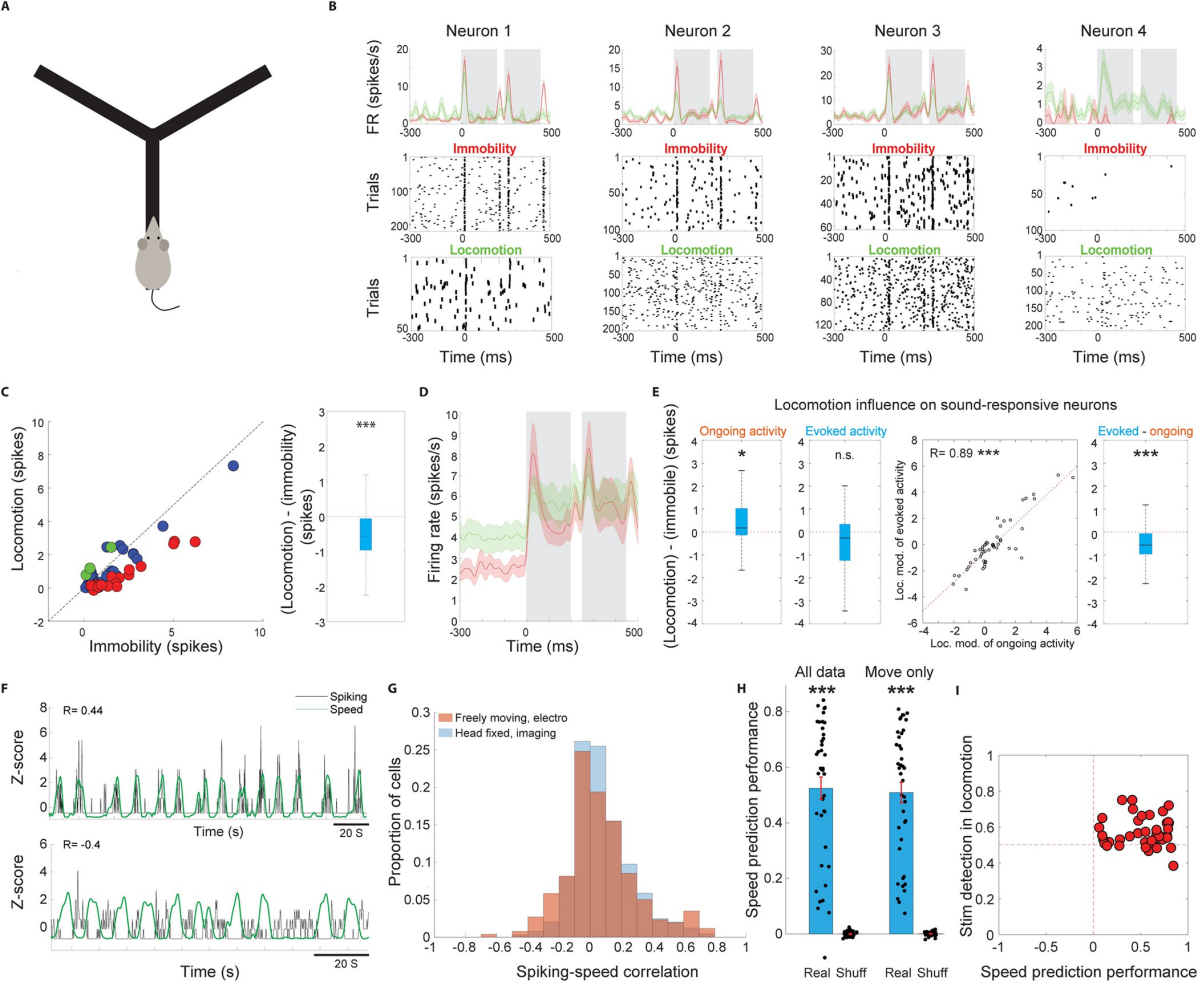
Integration of sound and locomotion in the freely moving rat. **(A)** Illustration of the experimental setup for electrophysiological recordings in freely moving rats. **(B)** Sound-triggered PSTHs from 4 example neurons. Sound presentation trials in which the animal was immobile (red) and running (green) were grouped separately. Neurons showed diverse patterns of modulation of sound-evoked responses during locomotion. **(C)** Left: Sound-evoked responses in immobility and locomotion across all target-sound responsive neurons. Red and green circles denote neurons that individually exhibited a significantly stronger and weaker response during immobility, respectively. Blue circles denote neurons that did not exhibit a significant difference. Right: The per-neuron difference in sound-evoked response between locomotion and immobility across all responsive neurons was significantly lower than 0 (*P* = 3.4 × 10^−5^, two-sided Wilcoxon signed-rank). For this and subsequent whisker plots, the central mark indicates the median, the bottom and top edges of the box indicate the 25th and 75th percentiles, respectively, and the whiskers extend to the most extreme data points not considered outliers. **(D)** Population-level PSTH across all target-sound responsive neurons during immobility (red) and locomotion (green). Solid lines and shaded areas indicate mean ± SEM. **(E)** Locomotion increased ongoing activity of sound-responsive neurons (left, *P* = 0.0144, two-sided Wilcoxon signed-rank test). Locomotion did not significantly modulate evoked activity (second from left, *P* = 0.2687, two-sided Wilcoxon signed-rank test). Locomotion influence on ongoing and evoked activity was correlated across neurons (second from right). The locomotion influence on evoked activity was significantly lower than that of ongoing activity, resulting in a net reduction in baseline-subtracted sound-evoked responses (right, *P* = 3.4 × 10^−5^, two-sided Wilcoxon signed-rank test). **(F)** Top: Z-scored spiking of an example neuron (black trace) overlaid on the Z-scored locomotion speed of the rat (green trace) during an example session. This neuron exhibited a correlation of R = 0.44 with locomotion speed across the session. Bottom: An example from a different neuron, showing a negative correlation with locomotion speed of R = −0.4. **(G)** Distribution of spiking-locomotion speed correlation values (orange). The parallel distribution from the imaging data (Fig 4D) is shown in light blue in the background as comparison**. (H)** Speed prediction performance, measured as the correlation values between the predicted and real locomotion speeds across ensembles. Shuffled values were derived by randomly shuffling the predicted speed values. **(I)** Stimulus detection in locomotion against speed prediction performance across ensembles. The data underlying this figure can be found in Fig 6 data at https://doi.org/10.6084/m9.figshare.23736831. PSTH, peri-stimulus time histogram.

Finally, we wished to test whether our findings of joint coding of locomotion speed and sound in head-fixed animals generalize to freely moving animals. To this end, we analyzed electrophysiological recordings from freely moving rats that were implanted with tetrodes in the AC [81]. Recordings were carried out as rats traversed a Y-shaped track for food reward delivered at reward wells (**Fig 6A**). In a pseudorandom approximately 25% of trials, following nose-poking in the home well rats were presented with series of chirp-pair sounds, which signaled that subsequent reward is delivered in the Sound well. Rats trained on this task for up to 15 days and reached good performance within 5 to 6 days [81]. We identified putative excitatory and fast-spiking interneurons based on spike waveform and firing rates (**S6A and S6B Fig**). We recorded a total of 248 putative excitatory neurons that had a sufficient number of responses in both immobility and locomotion to allow comparison. Of these, 21% (51/248) were significantly responsive to the target sound during immobility.

We first examined the effect of locomotion on baseline-subtracted sound-evoked spiking responses in freely moving rats by separating responses of putative excitatory neurons that occurred during immobility and locomotion. While individual neurons exhibited diverse influence by locomotion, across the population of target sound-responsive neurons, sound-evoked responses were significantly weaker during locomotion as compared to immobility (**Fig 6B and 6C**), consistent with our imaging data (**Fig 2E**) and previous studies [35,37]. Putative fast-spiking interneurons exhibited an even stronger suppression of sound-evoked responses during locomotion (**S6C Fig**).

We thus sought to test whether this locomotion-related decrease in baseline-subtracted sound-evoked responses could in part be due to increased baseline firing during locomotion as our imaging data in mouse indicated. Indeed, we found that ongoing activity, measured as the spike rate preceding stimuli presentations, was significantly higher during locomotion as compared to immobility across sound-responsive putative excitatory neurons (**Fig 6D and 6E**, left panel). Locomotion had no significant overall effect on spiking activity during the sound-evoked time window (**Fig 6E**, second panel), which differed from the elevation observed in the imaging data, likely due to differences in the targeted cortical layers across these datasets. Nevertheless, as in the imaging data, we observed a larger increase in ongoing activity than evoked activity, resulting in a net reduction in baseline-subtracted sound-evoked responses during locomotion (**Fig 6E**). These data demonstrate that, consistent with our head-fixed mouse data, increased ongoing activity during locomotion contributes to weaker sound-evoked responses in the freely moving rat as well.

To test whether increased ongoing activity during locomotion encodes locomotion speed in the freely moving rat, we examined correlations between continuous spiking activity and locomotion speed. We found similar results to the head-fixed mouse data, with the spiking activity of some neurons reliably tracking locomotion speed (**Fig 6F**). Across the population of putative excitatory neurons, the distribution of correlations between ongoing activity and locomotion speed was skewed to the right and highly similar to the distribution of the head-fixed data (**Fig 6G**). The spiking-speed correlation distribution of putative fast-spiking interneurons was significantly higher, though it is possible that this effect was at least partly due to their higher firing rates (**S6D Fig**). To further investigate the temporal relationship between locomotion speed and neural activity, we calculated the cross-correlation between these signals. We found that neural activity-speed cross correlation peaked at 0 second lag and decayed substantially even with a shift of 1 s, indicating that AC neural activity tracks locomotion speed with a rapid time constant (**S7 Fig**). Consistently, despite having a substantially lower number of simultaneously recorded putative excitatory neurons as compared to the imaging data (mean ± SEM electro: 4.5 ± 0.39, imaging: 29.3 ± 4.6), ensemble-level spiking activity could reliably predict locomotion speed (**Fig 6H**). Indeed, the number of neurons within an ensemble positively correlated with speed prediction performance (**S8 Fig**). Finally, we carried out a similar decoding analysis to that implemented on the imaging data and found that despite the low number of neurons per ensemble, many ensembles jointly coded for locomotion speed and sound in locomotion (**Fig 6I**). Interestingly, sound detection in locomotion showed a small but significant increase across days of training on this task while speed prediction did not significantly change across days (**S9 Fig**). Together, these findings suggest that encoding of locomotion speed and its integration with sound information is a robust feature of AC ensembles in the freely moving rat as well.

## Discussion

In this study, we tested the hypothesis that the AC plays a key role in auditory processing during locomotion and investigated the neural computation that it performs in this state. Using AC inactivation in behaving mice, we found that AC activity is required for sound-guided behavior during locomotion. Using two-photon calcium imaging in L2/3 of AC of head-fixed mice, we found that locomotion had a diverse but overall inhibitory influence on sound-evoked responses of individual neurons, which resulted in a mild but significant reduction in ensemble-level stimulus detection. Across ensembles, stimulus detection in immobility and locomotion were positively correlated, suggesting that sound processing across these states is supported by shared local neural populations in L2/3 of AC. Furthermore, we found that the net reduction in sound-evoked responses during locomotion are at least partly a result of increased ongoing neural activity, and importantly, that this ongoing activity robustly encoded the animal’s running speed. Thus, lower sound-evoked responses during locomotion reflected a tradeoff with the emergence of locomotion speed coding. Decoding analyses revealed that local neuronal ensembles of a few dozen neurons could jointly code locomotion speed and sound with high fidelity. Finally, we found consistent patterns of co-encoding of sound and locomotion speed in electrophysiologically recorded freely moving rats.

Previous studies have found that AC sound-evoked responses are on average weaker during locomotion as compared to immobility [35–39], a finding we have replicated here in both head-fixed mice and freely moving rats. Attenuation of responses to locomotion-associated self-generated sounds is well explained by corollary discharge, which acts to suppress responses to predictable sounds and enhance sensitivity to unpredictable sounds [38,40–43] (though see [44–46]). However, the functional benefit of the observed attenuation of AC responses to unpredictable external sounds during locomotion has remained elusive. This finding is particularly enigmatic given the critical need to be able to efficiently process external sounds and their associated meaning during locomotion for survival and the well-established role that AC plays in behavior- and context-dependent sound processing [22–34]. A proposed explanation for this finding is that during locomotion, neural computational resources shift from auditory to visual processing [35,36]. According to this proposal, weaker AC responses during locomotion reflect a reduced involvement of AC in sound processing in this state, in parallel to an enhancement of visual processing supported by increased responses in the visual cortex [47–49]. According to this model, reduced sound responses during locomotion reflect a functional attenuation of AC, possibly suggesting reliance on subcortical regions for sound processing in this state. However, the evolutionary and functional logic of this finding remains debated [37].

Our data strongly points at an alternative role of the AC in processing external sounds during locomotion. First, in contrast to the processing of simple sounds in immobility [55–63], our inactivation experiments show that sound processing during locomotion is strongly dependent on the AC (**Figs 1, S1 and S2**), arguing against a reduction of AC involvement in sound processing during locomotion. Second, both our imaging and electrophysiological data show that the locomotion-associated reduction in baseline-subtracted sound-evoked responses is at least partly a result of increased ongoing activity, which compresses the response dynamic range, rather than being a result of simple reduction in evoked firing rates (**Figs 3** and **6E**). Critically, rather than reflecting enhanced “noise,” the locomotion-associated elevated ongoing activity robustly encodes locomotion speed (**Figs 4, 6G and 6H**). While individual neurons showed heterogeneous locomotion-associated modulation of sound responses and ongoing activity, AC ensembles robustly jointly coded and integrated locomotion and sound (**Figs 5** and **6I**). Thus, we propose that rather than being inhibited during locomotion, AC ensembles explicitly encode it, alongside sound information, to provide a sound-in-motion signal.

Why is locomotion speed encoded in the AC? For both humans and other animals, the same sound can carry different meanings and appropriate motor responses depending on the precise locomotive state, requiring ongoing integration between sound and motion. For example, a person hearing a train passing a few feet in front of them may not show a behavioral response if the person is standing still, may slow down if they are walking slowly, or may jolt backwards if they are running. Similar audiomotor integration is required in rodents, for example, when fleeing a predator or hunting prey using sound. While one possibility is that in these scenarios, the auditory pathway would only represent sound and the integration with action would occur at a downstream integrative brain region, our data suggest that the AC itself may be that region. Moreover, recent findings suggest that robust representation of motor action and its integration with sensory information may be a common functional principle across sensory cortices. Specifically, although V1 responses are on average enhanced during locomotion, a number of studies have found that the influence of locomotion on visual cortical processing is better explained by sensory-motor integration than a general increase in gain. For example, one study found that locomotion modulates visual spatial integration by preferentially enhancing responses to larger visual objects [50]. An additional study found that V1 neurons are tuned to weighted combinations of locomotion speed and the speed of the incoming visual stimulus, giving rise to multimodal locomotion-visual representations in V1 [53]. Based on these and additional studies [54,82,83], it has been suggested that beyond simple modulation of response magnitude, a key function of V1 is to integrate visual and locomotion information in ways that inform action and navigation [84]. Although the influence of locomotion on the magnitude of stimulus-evoked responses in the visual and auditory cortices appear distinct, our findings suggest that the neural coding scheme reflecting joint representation and integration of locomotion and sensory cues is dominant in the AC as well and may reflect a general cortical functional principle.

In both our head-fixed mouse and freely moving rat data, locomotion speed coding emerged from individual neurons showing diverse but largely positive correlations between ongoing activity and locomotion speed (**Fig 4A–4E**). This finding differs from some previous studies, which found that ongoing activity in AC was suppressed during (and even just before) locomotion, due to input from the motor cortex and activity of local interneurons [35,36], a finding that would result in an overall negative correlation between neural activity and locomotion speed. However, our findings are consistent with other studies that observed an overall increase in ongoing activity during locomotion [33,37,39]. The discrepancy between these findings may be due to experimental differences that may result in targeting of functionally different neuronal subpopulations.

Locomotion is a complex behavior that is a result of coordinated motor activity, but it also involves changes in other factors such as arousal, motivation, attention, and effort. This raises the question of whether the findings described here—a reduction in magnitude of sound-evoked responses in parallel to the emergence of speed coding—are specific to locomotion or may be an indirect consequence of one of these factors. A reduction in the magnitude of AC sound-evoked responses is not unique to locomotion, and has also been observed, for example, as a result of increased behavioral engagement in the absence of locomotion [40,85], although the mechanisms may differ. However, we believe that locomotion speed coding by ongoing activity is unlikely to be a result of alternative factors. Our data shows that the spontaneous activity of many neurons tracks ongoing locomotion speed with high reliability even when excluding periods of immobility (**Figs 4H** and **6H**) and that neural activity tracks locomotion speed with a rapid time constant (**S7 Fig**). In contrast, while arousal level, attention, and motivation can all influence spontaneous activity in the AC [33,86–88] and typically increase in the transition from immobility to locomotion, there is no evidence that they rapidly track ongoing changes in locomotion speed. Specifically, previous studies that recorded pupil size as an indicator of arousal found that pupil size is larger during periods of locomotion relative to immobility, but it does not fluctuate rapidly enough to track locomotion speed [33,48,86,89,90]. Furthermore, ongoing firing rates were found to be higher during locomotion than during immobility for the same level of pupil-measured arousal [91]. Moreover, the similarity in our findings of locomotion speed coding in the mouse imaging data and the rat electrophysiology data, as well as the similarity in speed coding across the rat training days (**S9C Fig**), despite substantial differences in the motivational and attentional demands across these conditions, further reduce the likelihood that these factors substantially contribute to the findings. Finally, studies in other cortical areas also suggest distinct contributions of arousal and locomotion on cortical activity [48,90]. The contribution of physical effort is harder to dissociate from locomotion speed, as these behavioral attributes are strongly linked. To do this, future studies could use elevated surfaces or treadmills with varying degrees of resistance. An additional alternative is that the encoding of locomotion speed is linked to coding of sound source location [92–97]. As an animal runs faster through its environment, it experiences an increased level of change in sound source locations relative to itself, and this could be reflected in ongoing activity. In our mouse imaging data, animals were head fixed and thus sound source locations did not actually move relative to the animal, arguing against this possibility. However, we cannot fully rule out a contribution from secondary factors related to spatial hearing, such as increased spatial attention effort. To test this possibility, future studies could modulate the relationship between running speed and attended sound source location, for example, with an auditory virtual reality system [98].

Finally, the degree of stimulus specificity of the apparent response suppression during locomotion remains to be further explored. Indeed, locomotion-related attenuation of self-generated sounds is stimulus- and prediction-dependent [38,40–43]. In our imaging experiments, we found that responses to BBN, pure tones, and complex sounds were similarly suppressed during locomotion, though neither of these sounds carried immediate behavioral meaning to the animal and were presented at the same sound intensity. In our electrophysiological data, in which the target sound gained reward-location-predictive meaning across training, we found that locomotion-related responses strengthened (or were less attenuated) across days of training (**S9 Fig**). This suggests that locomotion-related attenuation of sound responses in the AC may be salience dependent. Moreover, sound intensity may strongly shape locomotion-related response modulation, with larger suppression of near-threshold sounds and lower suppression of louder sounds. Indeed, studies in humans have found that sound intensity and meaning influence the interaction between motor action and sound perception [99]. Future studies are required to further determine how locomotion modulates AC responses to sounds of varying attributes, such as intensity, behavioral relevance, valence, and sound source location.

## Methods

### Ethics statement

All procedures followed laboratory animal care guidelines, specifically animal protocol # PRO00009935, approved by the University of Michigan Institutional Animal Care and Use Committee and conformed to National Institutes of Health guidelines.

### Animals

A total of 32 male and female Thy1-GCaMP6f mice (C57BL/6J-Tg(Thy1-GCaMP6f)GP5.17Dkim/J, JAX stock No: 025393) between the ages of 12 to 23 weeks were used in this study (15 in the behavioral experiments and 17 in the two-photon experiments). Mice were kept on a reverse light cycle and all imaging and behavioral sessions were performed in the dark cycle.

Data from 4 Long–Evans male rats aged 4 to 5 months and weighing 450 to 550 g were also included in this study. Auditory cortical sleep data from these rats has been reported in an earlier study [81].

### Mouse surgery

Mice were anesthetized with Ketamine-Xylazine or isoflurane and implanted with a custom lightweight (<1 gr.) titanium head bar. For two-photon calcium imaging, the muscle overlying the right AC was removed and a 3 mm diameter glass cranial window was implanted over the right AC. For the cortical inactivation experiments, small bilateral craniotomies were drilled above the AC and either 2 mm or 3 mm length custom cannulas (Plastics One, Massachusetts, United States of America) were lowered into the AC. Mice received postop antibiotic ointment and Carprofen and were allowed to recover for at least 5 days before any imaging or behavioral sessions.

### Appetitive trace conditioning and AC inactivation

Mice were placed on water restriction 48 h prior to behavioral training and received ad libitum access to food. During training and testing, mice were placed in a custom built behavioral training box, in which they were head fixed on top of a rotatable plate with an accessible water reward port. A custom Arduino-based system that received input from a rotary encoder at the base of the plate allowed presenting sounds from a speaker placed approximately 10 cm in front of the animal in either immobility or locomotion.

Appetitive trace conditioning in locomotion: Animals were trained to associate a 1 s 8 kHz tone with subsequent water reward delivered after a delay of 1 s following sound termination, with sounds presented during locomotion. Specifically, mice were trained on a task in which sounds (followed by water reward) were presented exclusively in locomotion after the animal had run a distance that randomly varied across trials between 25 and 55 20ths of a full rotation. If the animal paused for longer than 2 s, then the trial was reset. Animals advanced to the testing phase only after they displayed consistent post-sound reward-predictive licking in locomotion for 2 consecutive days. Unrewarded catch trials (10% of trials) and immobility trials were used to validate sound-triggered and locomotion-specific licking, respectively. Similar to the immobility conditions, mice were first tested following bilateral infusion of 750 nL PBS solution into AC and 24 h later following infusion of 750 nL muscimol (*1 μg/μl*).

Appetitive trace conditioning in immobility: Animals were trained to associate a 1 s 8 kHz tone with subsequent water reward delivered after a delay of 1s following sound termination. Sounds (followed by water rewards) were presented following a period of continuous immobility that randomly varied across trials between 5 and 10 s. If the animal ran, the immobile duration counter was reset. Animals advanced to the testing phase only after they displayed consistent post-sound reward-predictive licking in locomotion for 2 consecutive days. Animals were tested following bilateral infusion of 750 nL PBS solution into AC, and 24 h following PBS infusions animals were tested following bilateral infusions of 750 nL muscimol (1 μg/μl). Unrewarded catch trials (10% of trials) were used to validate sound-triggered licking.

To quantify the association between sound and subsequent water reward, we quantified the degree of increased licking in the 1 s window following sound termination and before reward delivery (0 to 1,000 ms from sound offset) relative to the pre-sound baseline lick rate ((−1,500)–(−500) ms from sound onset). To this end, we defined a “predictive lick index” as the across-trials average difference between the number of licks in the post-sound window and that of the pre-sound window.

### Two-photon imaging

Imaging was carried out as described previously [100,101]. During imaging sessions, mice were placed on a rotating plate while being head fixed under the microscope objective. Imaging was carried out while the head of the animal was straight, with the objective tilted using an orbital nosepiece to allow optical access to the AC. Mice were allowed to initiate movement at their leisure. Imaging was performed using an Ultima IV two-photon microscope (Bruker), a pulsed tunable laser (MaiTai eHP DeepSee by Spectra Physics) providing excitation light at 940 nm and 16× or 40× water-immersion objectives (Nikon). Images (256 × 256 pixels) were acquired using galvanometric mirrors at approximately 3 Hz to optimize signal quality and cell separation. The microscope was placed in an enclosed chamber in a dark, quiet room. Neurons were imaged at depths of 150 to 350 μm, corresponding to cortical L2/3.

During imaging sessions, the mouse’s behavior was video recorded using an infrared camera, which was synchronized offline with the imaging data acquisition. Locomotion and immobility were determined offline using semi-automatic movement-detection MATLAB code with manual thresholding and supervision. In addition, in most imaging sessions, a rotary encoder was positioned at the base of the rotating plate allowing to acquire continuous locomotion speed. In a given daily imaging session, responses of the same neurons were imaged to multiple sound protocols. Different neuronal ensembles in the same mice were typically imaged on separate days.

Auditory stimuli were delivered via an open-field magnetic speaker (MF1, Tucker Davis Technologies) at 75 dB. The broadband noise bursts protocol consisted of 45 repeats of 1 s white noise bursts with an interstimulus interval of 3 ± 1 s. The sound-masking sessions consisted of continuous presentation of broadband noise at 80 dB. The pure tone protocol (**S3 Fig**) consisted of 3 randomly shuffled pure tones (2 kHz, 4 kHz, and 8 kHz), of 20 repeats, with a duration of 1 s and an interstimulus interval of 3 ± 1 s. The complex sound protocol (**S3 Fig**) consisted of 4 randomly shuffled complex sounds (cricket, sparrow, scratch, and water), with 20 or 9 repeats per stimulus. Complex stimuli duration ranged from 0.2 to 0.5 s, padded with 0.8 to 0.2 s of silence to create 1 s long stimuli frames and an interstimulus interval of 3 ± 1 s.

### Imaging data preprocessing and analysis

Daily imaging data of the same ensemble across multiple sound protocols was concatenated and then preprocessed using the open source Suite2P software [102] for movement correction and neuronal ROI detection within the ensemble. Neural data, sound stimuli, and locomotion speed signals were aligned.

Data analysis was performed using custom software written in Matlab (MathWorks).

Relative change in fluorescence (ΔF/F) across time (t) was calculated for each detected cell as $\frac{F(t)-median(F)}{median(F)}$, where F(t) is the mean brightness of the cell’s pixels at time t.

For determination of BBN-responsiveness of individual neurons and quantification of activity in the pre-stimulus time window (Ongoing) and stimulus time window (Evoked), the mean ΔF/F was taken across 1 to 4 samples preceding stimulus onset (corresponding to approximately −1.2 to 0 s), and 1 to 4 samples following stimulus onset (corresponding to approximately 0 to 1.2 s), respectively. A cell was determined as BBN-responsive if ΔF/F during the stimulus time window was significantly higher than during the pre-stimulus time window using a one-tailed paired *t* test at *P* < 0.05 across all immobile trials. Ongoing activity levels in immobility/locomotion in the presence of background masking noise were quantified as the average ΔF/F across all time points of immobility/locomotion in the session.

A difference in BBN-evoked response magnitude between immobility and locomotion was determined using an unpaired two-sided *t* test (at *P* < 0.05) of the response magnitudes during the immobility and locomotion trials. Neurons with 8 or fewer responses in either state (immobility/locomotion) were excluded from immobility/locomotion comparisons. To determine a difference in the influence of locomotion on responses to tones and complex sounds, locomotion and immobility trials of each stimulus were compared separately.

For calculating ΔF/F-locomotion speed correlations and speed prediction, the daily locomotion speed was smoothed using a 6-sample (approximately 2 s) moving average filter. The ΔF/F-locomotion speed correlation was calculated as the Pearson correlation between the continuous ΔF/F trace of each neuron and the animal’s locomotion speed.

Speed prediction was carried out using cross-validated GLMs on the day’s ensemble continuous activity patterns and locomotion speed. For a given ensemble, the data included the daily continuous locomotion speed and ΔF/F traces of all cells. ΔF/F traces of each cell were smoothed using a 3-sample (approximately 1 s) moving average filter. Locomotion speed in cm/s was log-transformed using log(*speed*+1). In the model training phase, a random half of the daily sample points (“training set”) of locomotion and corresponding ensemble ΔF/F values were used to train a GLM. In the test phase, the model used the remaining ensemble ΔF/F values (“test set”) to predict the corresponding (log) locomotion speeds. Prediction performance was quantified by the Spearman correlation between the predicted speeds and the real speeds. This procedure was repeated 200 times and the correlation values averaged across repeats to yield the final prediction performance. Repeats in which the test set included fewer than 10 nonzero speed values were excluded.

Stimulus detection during locomotion was quantified using cross-validated SVM analyses. Only ensembles with more than 10 cells and 12 trials in both immobility and locomotion were included in the decoding analyses. Data consisted of all ensemble activity patterns before BBN presentation (i.e., across-trials ensemble activity in the pre-stimulus time windows) and during BBN presentation (i.e., across-trials ensemble activity in the stimulus time windows) that occurred during locomotion. An SVM model was constructed on this data and a 10-fold cross validation was used to estimate the ability of the ensemble to discriminate between the pre-stimulus and stimulus ensemble activity patterns. Detection performance was defined as the across-fold average of percent correct predictions. To estimate significance of prediction, this procedure was performed 200 times on shuffled data identity and significant detection was determined if detection performance was higher than 95% of shuffles. Discrimination of locomotion state (immobility/locomotion) was carried out in a similar manner, but using (1) The ensemble activity patterns during the pre-stimulus time windows in immobility and (2) the pre-stimulus time windows in locomotion, as the data to be discriminated.

Analysis of the electrophysiology data was carried out similarly to the imaging data, but using spike counts instead of ΔF/F as the neural measure and using a stimulus response time window of 1 to 450 ms and a pre-stimulus time window of −450 to 0 ms relative to sound onset. Immobility was determined as speed < = 4 cm/s; neurons with 10 or fewer responses in either state (immobility/locomotion) were excluded from immobility/locomotion comparisons. A minimum of 20 trials in both immobility and locomotion were required for inclusion in the decoding analyses. Spiking-speed correlations were calculated by binning spiking and speeds into 200 ms bins and calculating the Spearman correlation. Speed prediction of movement-only data excluded all data with movement slower than 0.65 cm/s.

### Rat pretraining, surgery, and electrophysiological recordings

The rat behavioral and surgery procedures have been described previously [81,103]. Briefly, after habituation to daily handling over several weeks, rats were pretrained to run on an E-shaped raised track for liquid food rewards (sweetened condensed milk). Rats were then implanted with a microdrive array with 21 independently moveable tetrodes (groups of 4 twisted 12.5 μm nichrome wires assembled in a bundle). Seven tetrodes were targeted to the left primary AC (−4.8 mm AP, 5.5 mm ML, 25° lateral from midline). Other tetrodes targeted left dorsal CA1 region of the hippocampus and left PFC, but these data are not included here. Over the course of 2 weeks following implantation, AC tetrodes were advanced gradually and responses to sound stimuli were used to validate approach to primary AC. Based on histological sections and the ventro-lateral angle of parallel tetrodes targeting the AC, the rat data likely includes neurons from a mixture of layers from 2 to 5.

Data were collected using the NSpike data acquisition system (L.M. Frank and J. MacArthur, Harvard Instrumentation Design Laboratory). Spike data were sampled at 30 kHz, digitally filtered between 300 Hz and 6 kHz (two-pole Bessel for high and low pass), and threshold crossing events were saved to disk (40 samples at 30 kHz). Individual units (putative single neurons) were identified by clustering spikes using peak amplitude, principal components and spike width as variables (MatClust). Behavior sessions were recorded with an overhead monochrome CCD camera (30 fps), and the animal’s position and speed were detected using an infrared light emitting diode array with a large and a small cluster of diodes attached to the preamps.

Approximately 14 d after implantation, animals were introduced to the Y-track and data gathering commenced. Animals were trained on the Y-track for 10 to 12 d in 3 to 4, 20-min training sessions per day with interleaving 20- to 30-min sleep sessions in the rest box. Data for each neuron was pooled across daily sessions. During training sessions, sweetened condensed milk rewards were automatically delivered in food wells triggered by animal’s nose-poke crossing of an IR beam. Rats initiated each trial by a nose-poke in the home well and receiving a reward. In approximately 75% of trials, the next reward was delivered in the silent well if the rat nose-poked there. In a pseudorandom approximately 25% of trials (sound trials separated by 2 to 5 silent trials), 5 s after nose-poking in the home arm, a target sound series was emitted from a speaker, indicating that the next reward would be delivered in the sound well if the rat next nose-poked there. The speaker was placed at the end of the sound arm in the first days of training and moved to the center junction after rats displayed consistent correct choices in more than ~70% of trials. The target sound was a pair of upward chirps, consisting of one 200-ms chirp with frequency modulated from 3 to 4 kHz, an interchirp interval of 50 ms, and a second 200-ms chirp with frequency modulated from 9 to 12 kHz. The series of target sounds was presented at 1 Hz and stopped after 12 s or once the rat made a correct or incorrect choice by a nose-poke in one of the wells. The sound-evoked responses analyzed in Fig 6 include sounds in which the spatial orientation and distance between the animal and the speaker varied based on the animal’s behavior. Thus, the data contains variability of effective intensity and spatial orientation of the sound source. Reward amount in the sound well was double the reward amount in the home or silent well. Following the Y-track training days, 2 rats continued to perform the same task on a W-shaped track for an additional 3 days.

## Supporting information

S1 Fig AC inactivation alters locomotion speed following sound presentation. **(A)** Sound-triggered PSTH showing changes in locomotor activity following sound presentation in locomotion in PBS (blue) and MUS (orange). **(B)** (Left) No significant difference in average step rate at sound onset for animals under PBS and MUS conditions (*P* = 0.569, two-sided Wilcoxon signed-rank test). (Right) Significant difference in the average step rate preceding reward delivery for animals under PBS and MUS conditions (*P* = 0.0078, two-sided Wilcoxon signed-rank test). **(C)** Relative decrease in locomotion speed following sound onset and preceding reward delivery is significantly greater in PBS compared to MUS conditions (*P* = 0.0078, two-sided Wilcoxon signed-rank test). The data underlying this figure can be found in S1 Fig data at https://doi.org/10.6084/m9.figshare.23736831.
(PDF)

S2 Fig Auditory cortical inactivation does not induce a significant impairment in sound-triggered reward-predictive licking in immobility. **(A–C)** Similar to Fig 1A–1C for immobility conditions. C: *P* = 0.46875, signed-rank test. **(D)** Calculation of predictive lick index in immobility and locomotion using an alternative lick window of 0–2 s. Immobility: *P* = 0.15625; Locomotion: *P* = 0.0391, signed-rank test. The data underlying this figure can be found in S2 Fig data at https://doi.org/10.6084/m9.figshare.23736831.
(PDF)

S3 Fig Locomotion influence on AC responses to tones and complex sounds. Baseline-subtracted sound-evoked responses in immobility and locomotion for tones (top) and complex sounds (bottom). Graphical conventions same as Fig 1E. While individual responses showed diversity in locomotion-related influence, population-level responses to both tones and complex sounds were significantly reduced during locomotion (Tones: *P* = 3.7 × 10^−5^, Complex sounds: *P* = 0.0402, two-sided Wilcoxon signed-rank test). The data underlying this figure can be found in S3 Fig data at https://doi.org/10.6084/m9.figshare.23736831.
(PDF)

S4 Fig Influence of locomotion on ongoing activity under masking noise conditions. The data underlying this figure can be found in S4 Fig data at https://doi.org/10.6084/m9.figshare.23736831.
(PDF)

S5 Fig Locomotion influence on all neurons (including sound-unresponsive). Same layout as Fig 3B and 3D. Left, *P* = 7.05 × 10^−27^; middle, *P* = 4.11 × 10^−29^; right, *P* = 0.0693, two-sided Wilcoxon signed-rank test. The data underlying this figure can be found in S5 Fig data at https://doi.org/10.6084/m9.figshare.23736831.
(PDF)

S6 Fig Locomotion modulation of putative fast-spiking interneurons. **(A)** Detection of putative FS interneurons (red) based on spike width and firing rate. **(B)** Waveforms of putative FS (red) and RS (black) neurons. **(C)** Influence of locomotion on sound-evoked responses of putative FS interneurons (as in Fig 2E). **(D)** Distribution of spiking-speed correlation for putative FS interneurons (orange) over the distribution for putative RS neurons (as in Fig 4D). The distribution of FS neurons was significantly higher than that of RS neurons (*P* = 0.0055, rank-sum test). There was no significant correlation between firing rate and spiking-speed correlation within putative excitatory neurons (R = −0.037, *P* = 0.557) or within putative FS interneurons (R = −0.075, *P* = 0.62). **(E)** Speed prediction performance of putative RS and FS single neurons. Prediction of FS neurons showed a higher trend, though this did not reach significance (*P* = 0.1036, rank-sum test). The data underlying this figure can be found in S6 Fig data at https://doi.org/10.6084/m9.figshare.23736831.
(PDF)

S7 Fig Auto correlation of speed (purple) and cross-correlation of speed and spiking (green) across the electrophysiology dataset, calculated across all data where the speed-spiking correlation was > = 0.3. Inset shows enlarged view of the highlighted area. The data underlying this figure can be found in S7 Fig data at https://doi.org/10.6084/m9.figshare.23736831.
(PDF)

S8 Fig Relationship between the number of cells in an ensemble and speed prediction performance in the electrophysiological data. The data underlying this figure can be found in S8 Fig data at https://doi.org/10.6084/m9.figshare.23736831.
(PDF)

S9 Fig Sound detection in locomotion **(A)**, sound detection in immobility **(B)** and speed coding **(C)** across training days for the electrophysiology data. The data underlying this figure can be found in S9 Fig data at https://doi.org/10.6084/m9.figshare.23736831.
(PDF)

## Abbreviations

AC: auditory cortex
BBN: broadband noise
GLM: generalized linear model
MUS: muscimol
PBS: phosphate buffer solution
PSTH: peri-stimulus time histogram
SVM: support vector machine
