# Supplementary figures and images for: Auditory cortex ensembles jointly encode sound and locomotion speed to support sound perception during movement
Source: PLoS Biol. 2023 Aug 31;21(8):e3002277. doi: 10.1371/journal.pbio.3002277 (PMC10499203; doi:10.1371/journal.pbio.3002277)

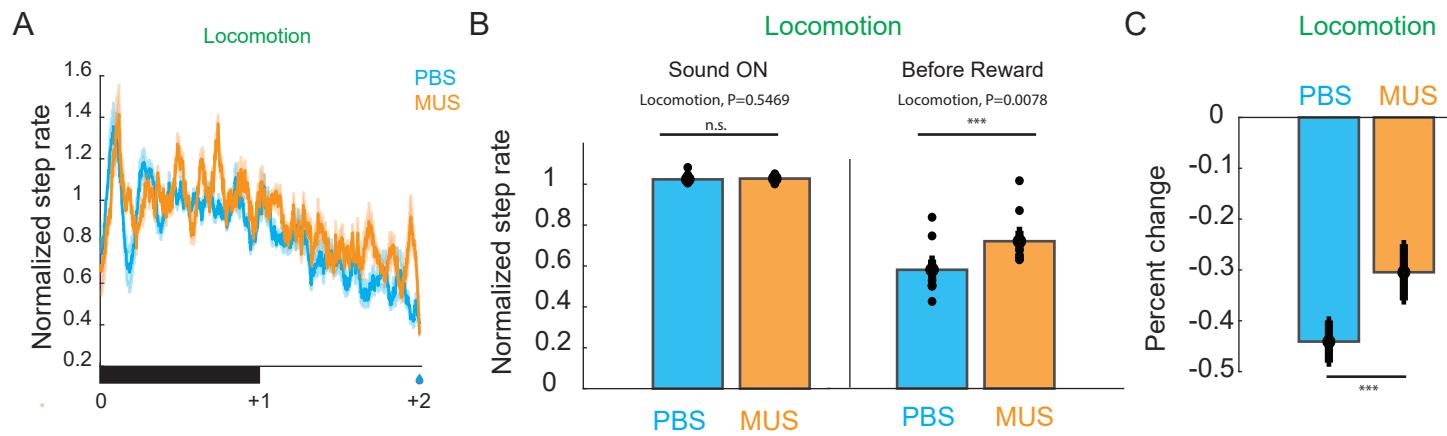

Supplement: S1 Fig — (A) Sound-triggered PSTH showing changes in locomotor activity following sound presentation in locomotion in PBS (blue) and MUS (orange). (B) (Left) No significant difference in average step rate at sound onset for animals under PBS and MUS conditions (P = 0.569, two-sided Wilcoxon signed-rank test). (Right) Significant difference in the average step rate preceding reward delivery for animals under PBS and MUS conditions (P = 0.0078, two-sided Wilcoxon signed-rank test). (C) Relative decrease in locomotion speed following sound onset and preceding reward delivery is significantly greater in PBS compared to MUS conditions (P = 0.0078, two-sided Wilcoxon signed-rank test). The data underlying this figure can be found in S1 Fig data at https://doi.org/10.6084/m9.figshare.23736831. (PDF) [file pbio.3002277.s001.pdf]

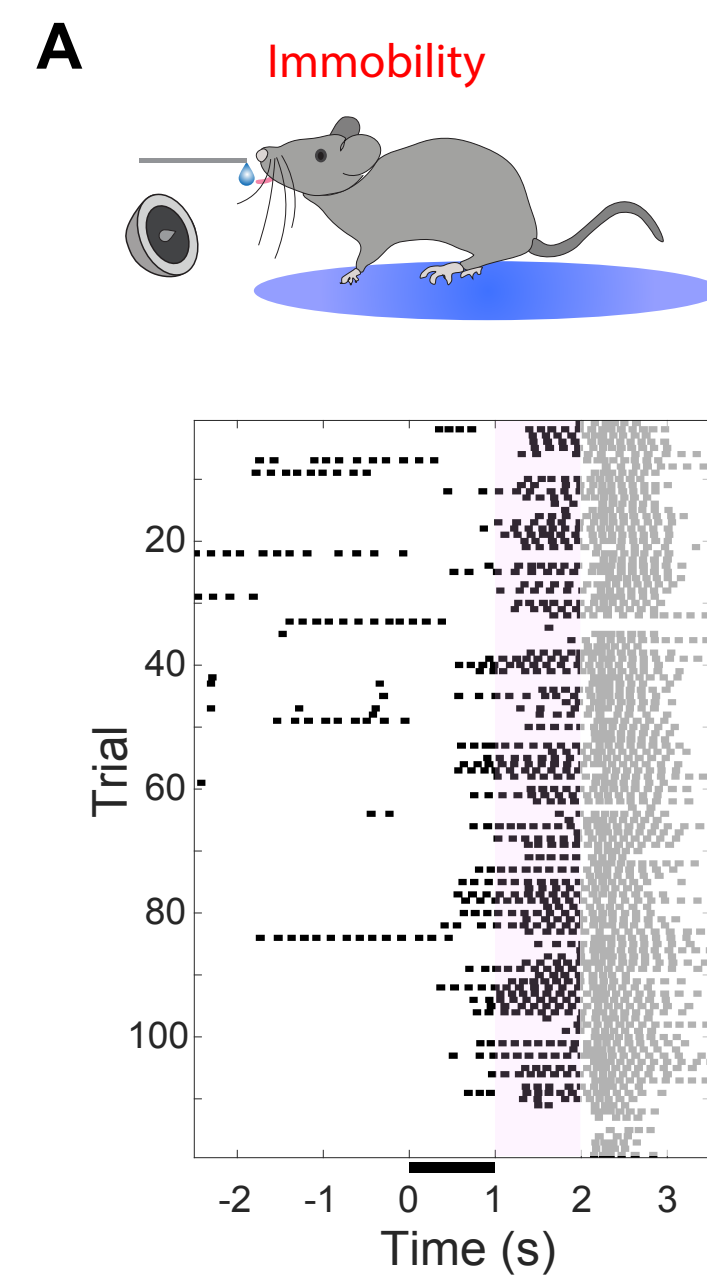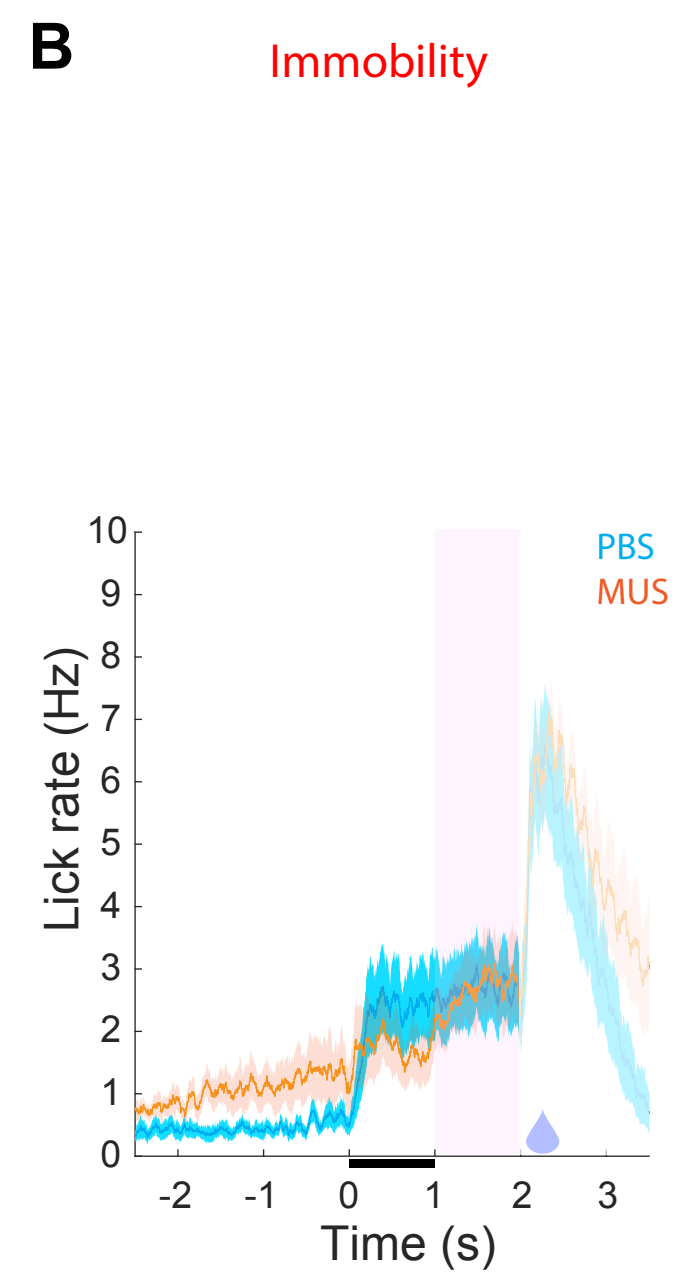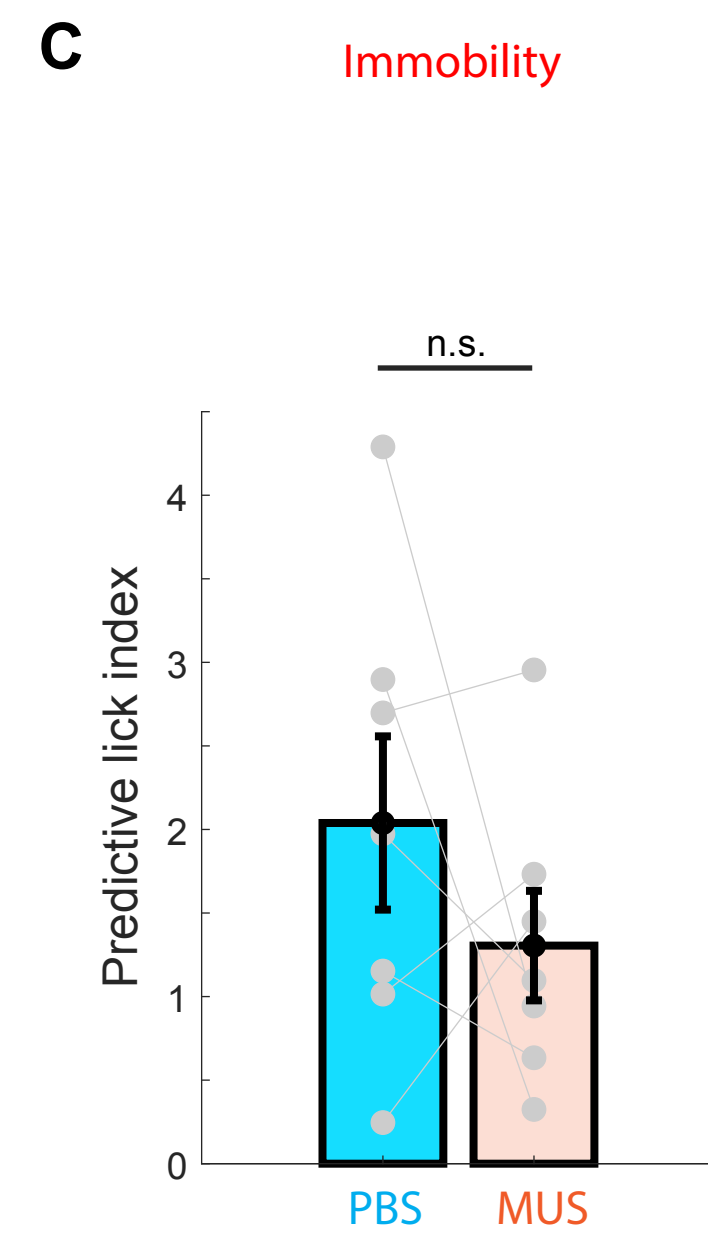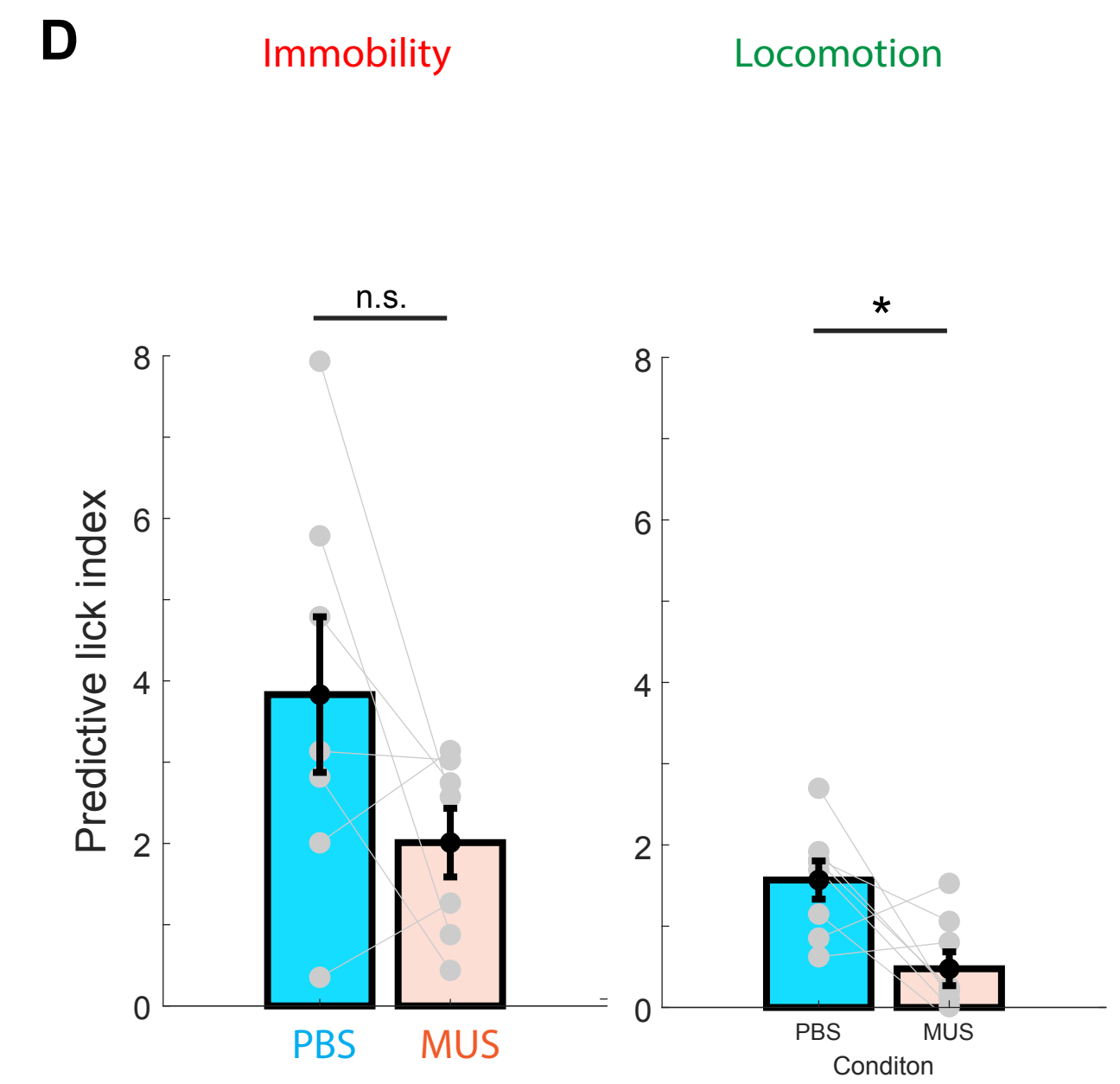

Supplement: S2 Fig — (A–C) Similar to Fig 1A–1C for immobility conditions. C: P = 0.46875, signed-rank test. (D) Calculation of predictive lick index in immobility and locomotion using an alternative lick window of 0–2 s. Immobility: P = 0.15625; Locomotion: P = 0.0391, signed-rank test. The data underlying this figure can be found in S2 Fig data at https://doi.org/10.6084/m9.figshare.23736831. (PDF) [file pbio.3002277.s002.pdf]

## Tones

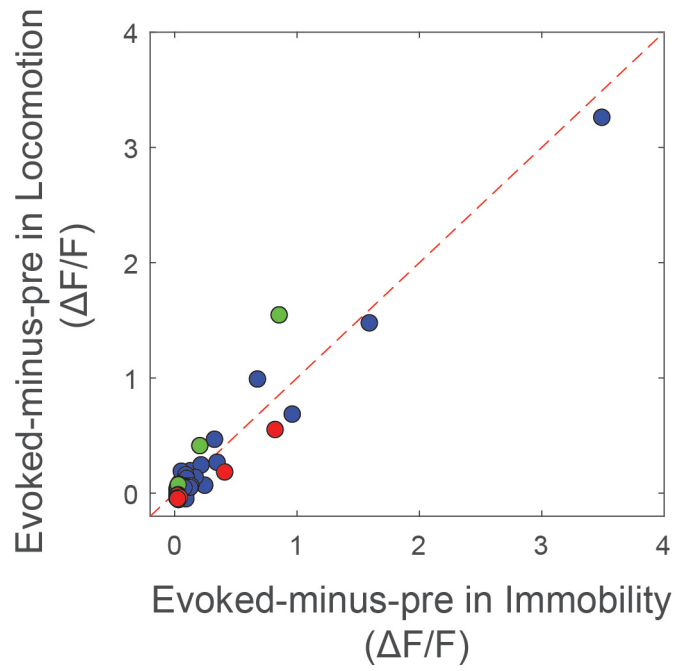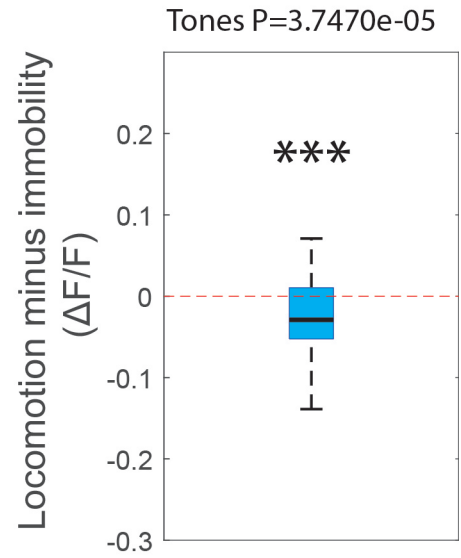

## Complex sounds

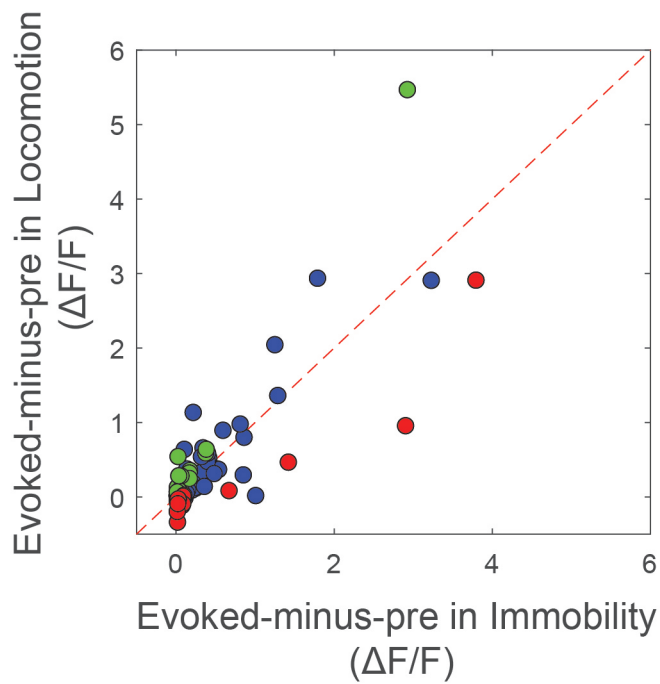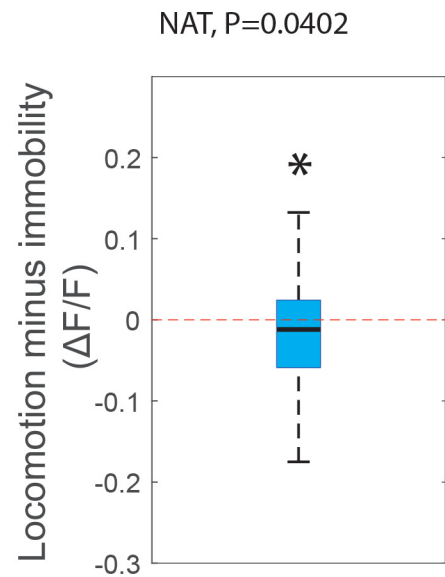

Supplement: S3 Fig — Baseline-subtracted sound-evoked responses in immobility and locomotion for tones (top) and complex sounds (bottom). Graphical conventions same as Fig 1E. While individual responses showed diversity in locomotion-related influence, population-level responses to both tones and complex sounds were significantly reduced during locomotion (Tones: P = 3.7 × 10^−5^, Complex sounds: P = 0.0402, two-sided Wilcoxon signed-rank test). The data underlying this figure can be found in S3 Fig data at https://doi.org/10.6084/m9.figshare.23736831. (PDF) [file pbio.3002277.s003.pdf]

## Ongoing activity in masking noise

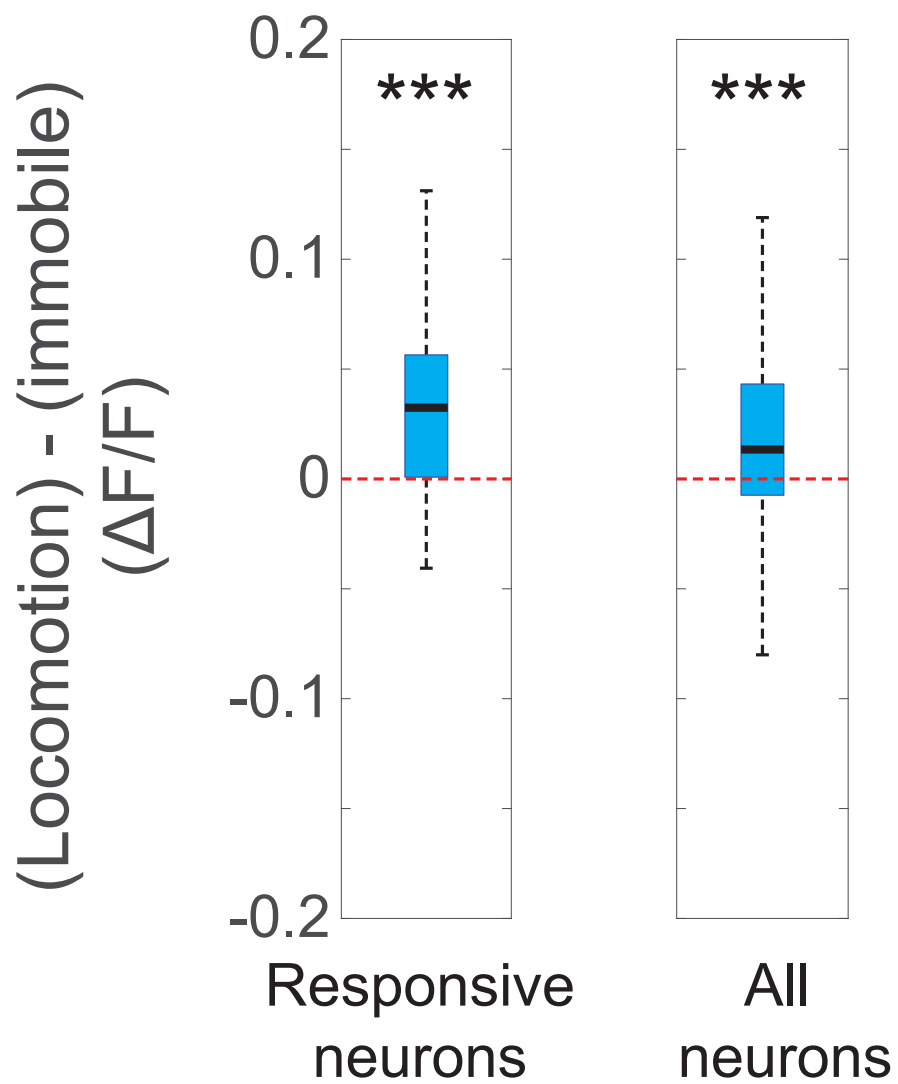

Supplement: S4 Fig — The data underlying this figure can be found in S4 Fig data at https://doi.org/10.6084/m9.figshare.23736831. (PDF) [file pbio.3002277.s004.pdf]

## Locomotion influence on all neurons

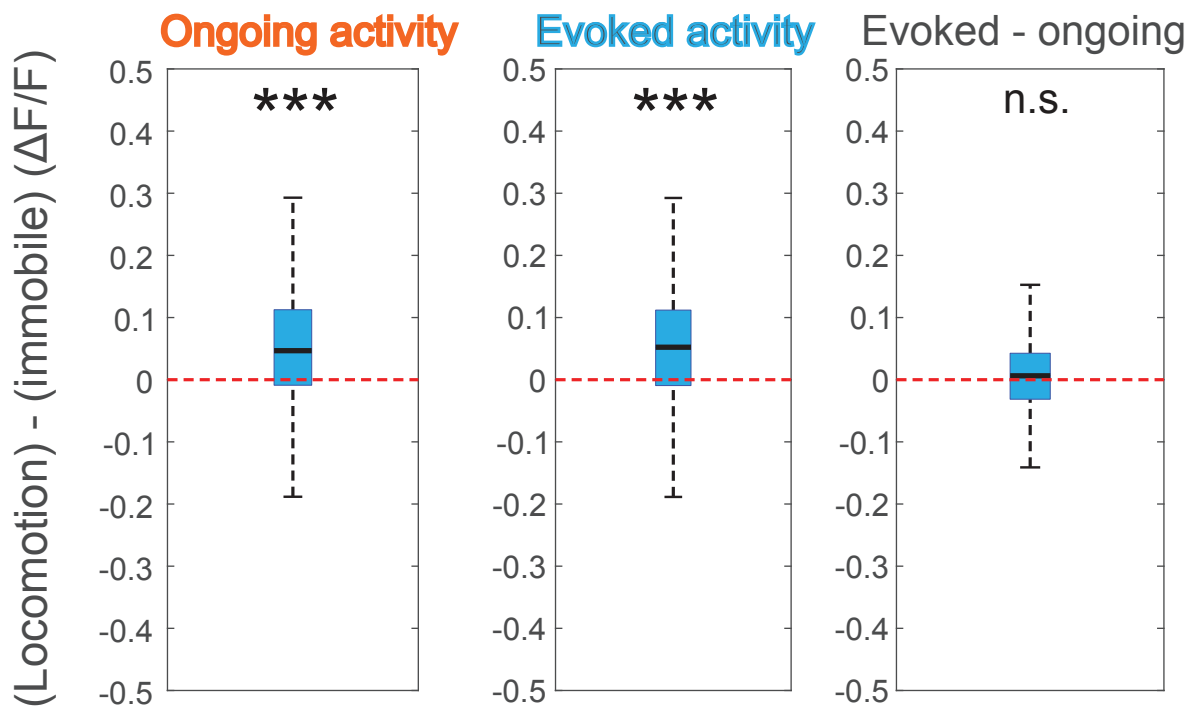

Supplement: S5 Fig — Same layout as Fig 3B and 3D. Left, P = 7.05 × 10^−27^; middle, P = 4.11 × 10^−29^; right, P = 0.0693, two-sided Wilcoxon signed-rank test. The data underlying this figure can be found in S5 Fig data at https://doi.org/10.6084/m9.figshare.23736831. (PDF) [file pbio.3002277.s005.pdf]

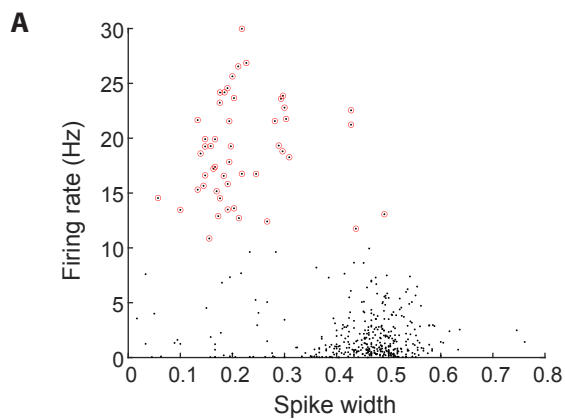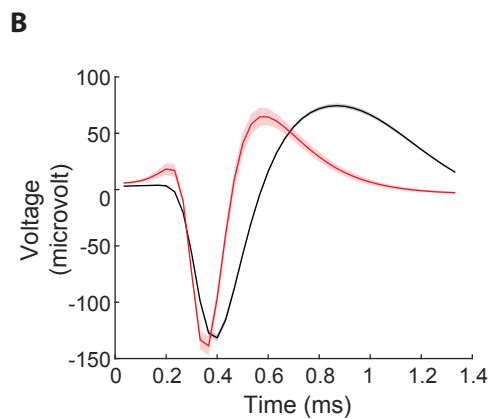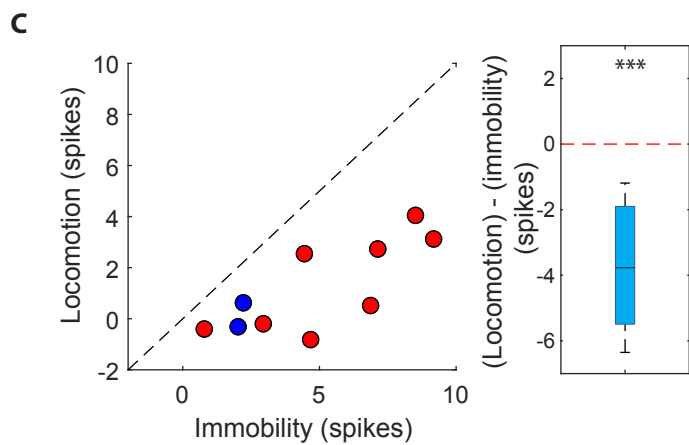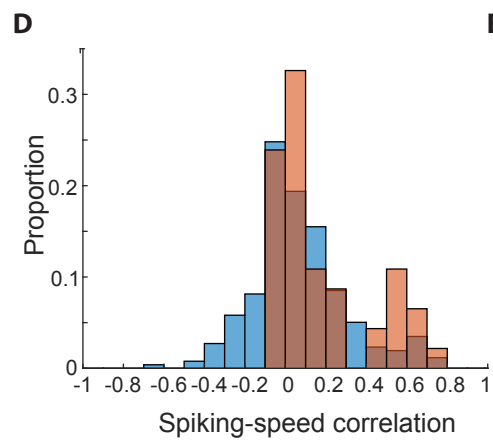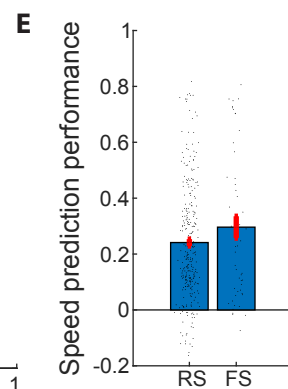

Supplement: S6 Fig — (A) Detection of putative FS interneurons (red) based on spike width and firing rate. (B) Waveforms of putative FS (red) and RS (black) neurons. (C) Influence of locomotion on sound-evoked responses of putative FS interneurons (as in Fig 2E). (D) Distribution of spiking-speed correlation for putative FS interneurons (orange) over the distribution for putative RS neurons (as in Fig 4D). The distribution of FS neurons was significantly higher than that of RS neurons (P = 0.0055, rank-sum test). There was no significant correlation between firing rate and spiking-speed correlation within putative excitatory neurons (R = −0.037, P = 0.557) or within putative FS interneurons (R = −0.075, P = 0.62). (E) Speed prediction performance of putative RS and FS single neurons. Prediction of FS neurons showed a higher trend, though this did not reach significance (P = 0.1036, rank-sum test). The data underlying this figure can be found in S6 Fig data at https://doi.org/10.6084/m9.figshare.23736831. (PDF) [file pbio.3002277.s006.pdf]

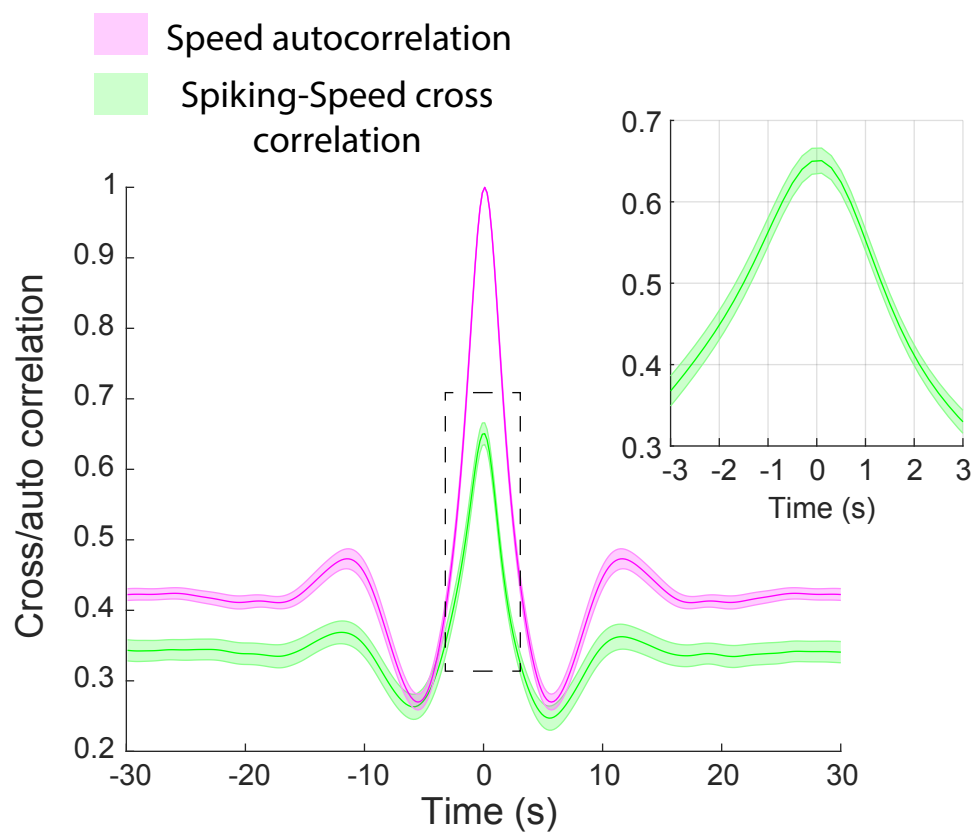

Supplement: S7 Fig — Inset shows enlarged view of the highlighted area. The data underlying this figure can be found in S7 Fig data at https://doi.org/10.6084/m9.figshare.23736831. (PDF) [file pbio.3002277.s007.pdf]

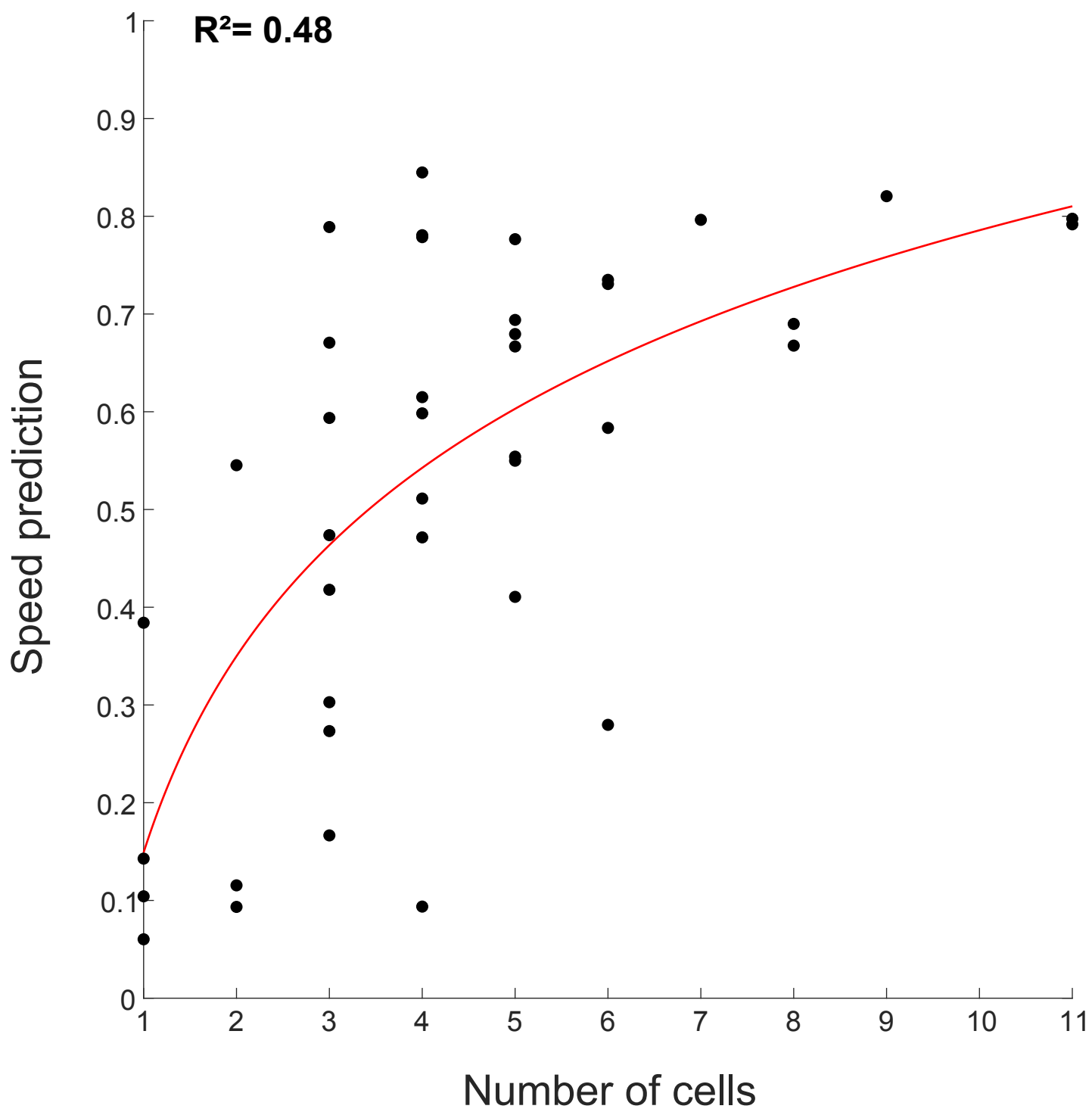

Supplement: S8 Fig — The data underlying this figure can be found in S8 Fig data at https://doi.org/10.6084/m9.figshare.23736831. (PDF) [file pbio.3002277.s008.pdf]

**A**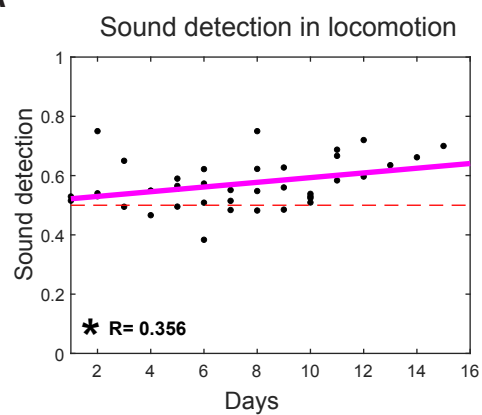**B**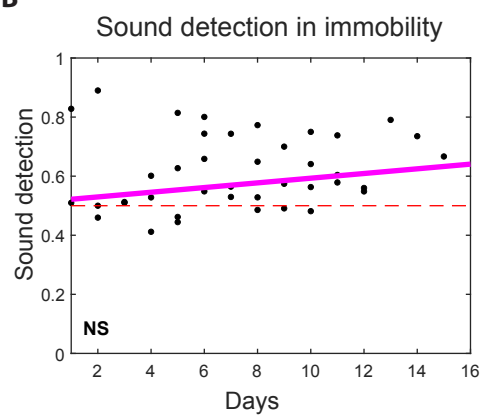**C**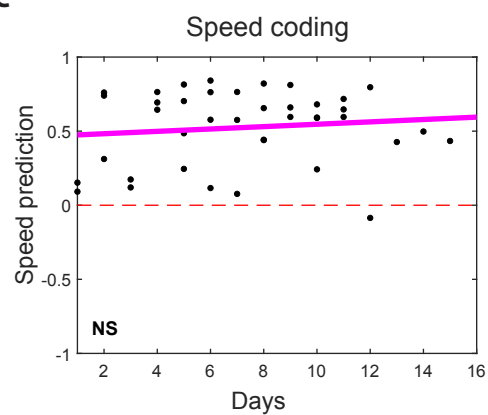

Supplement: S9 Fig — Sound detection in locomotion (A), sound detection in immobility (B) and speed coding (C) across training days for the electrophysiology data. The data underlying this figure can be found in S9 Fig data at https://doi.org/10.6084/m9.figshare.23736831. (PDF) [file pbio.3002277.s009.pdf]
